# T-Cell Receptor Single-Chain Antibody IgG1-Fc Fusion Proteins as Bispecific Engagers for Natural Killer and T Cells

**DOI:** 10.3390/cells15171545

**Published:** 2026-08-27

**Authors:** Annkathrin C. Teschner, Márcia Gonçalves, Marten Meyer, Inka Zörnig, Dirk Jäger, Frank Momburg

**Affiliations:** 1 Clinical Cooperation Unit Applied Tumor Immunity, German Cancer Research Center (DKFZ), Im Neuenheimer Feld 280, 69120 Heidelberg, Germany; annkathrin.teschner@pei.de (A.C.T.);; 2 Department of Medical Oncology, Heidelberg University Hospital, Heidelberg University, 69120 Heidelberg, Germany

**Keywords:** peptide/MHC-I complexes, T cell receptor (TCR), bispecific antibodies, TCR-based NK cell engager, ADCC, TCR-based T cell engager, T cell costimulation, tumor immunology

## Abstract

**Highlights:**

**What are the main findings?**

- Novel trifunctional NK cell engagers have been developed that combine the ADCC-eliciting function of IgG1 Fc parts and NK cell-targeting mini antibodies with the capacity of T cell receptors to specifically recognize intracellular peptide antigens presented by MHC molecules
- Antibody-like bivalent TCR/anti-CD3ε-based T cell engagers have been engineered.

**What are the implications of the main findings?**

- Redirection of NK cells toward peptide/MHC-I complexes presented by tumor cells via TCR-based NK cell engagers expands the therapeutic repertoire of tumor immunologists.
- The Ig-like TCR-based T cell engager format complements existing formats of smaller size with a format that may exhibit advantageous pharmacologic properties.

**Abstract:**

Soluble variants of recombinant T cell receptors (TCRs) have become attractive tools for the retargeting of cytotoxic T cells toward intracellular tumor or viral antigens by combining them with CD3-binding antibodies in bispecific T cell engagers; however, TCR-based NK cell engagers have not been studied so far. Here, we developed TCR-based bispecific agents for the redirection of NK cells utilizing a bivalent IgG1-like format. Trifunctional NK engagers included an Fc part with enhanced binding to FcγRIII/CD16A and single-chain (scFv) antibodies recognizing either NKp46 or CD16A, activating NK cell receptors. HCMV pp65/HLA-A2 reactive TCR-scFv-Fc fusion proteins incorporating an affinity-matured TCR and anti-NKp46 scFv activated peripheral blood NK cells and induced cytotoxicity in an antigen-specific manner. For T cell redirection, TCR-scFv-Fc fusion proteins included an scFv antibody recognizing CD3ε. Compared with NK cell engagers, T cell engagers showed similar sensitivity but lower peptide selectivity. For two TCRs recognizing melanoma-associated peptides, affinity-matured TCR-scFv-Fc fusion proteins enabled NK and T cell redirection and activation, and killing of tumor target cells loaded with exogenous peptides. Our results expand the versatility of the soluble TCR technology to NK cell engagers; however, they still require improvement in sensitivity to target tumor cells with low peptide/MHC-I complex densities.

## 1. Introduction

In the last decades, antibodies and antibody-like compounds have been demonstrated as a potent and versatile tool in cancer immunotherapy [1,2,3,4,5]. Their designated mode of action can range from blocking specific receptor–ligand interactions important for tumor growth, promoting immune cell activation by activating or inhibiting specific receptor pathways, delivering toxic payloads, or promoting tumor cell recognition and killing by bridging immune and tumor cells using bispecific molecules. In past years, bispecific immune cell engagers have gained increasing interest and have been developed in manifold forms for a variety of tumor targets [1,2]. Most bispecific antibodies (bsAb) have been designed to recruit cytotoxic T cells by bridging a tumor-associated antigen (TAA) to the T cell receptor (TCR) complex component CD3ε, allowing for T cell activation independent of TCR specificity [1,2,6,7], and ten anti-TAA–anti-CD3ε bsAb have been approved for the treatment of cancer until present [7,8].

While natural killer (NK) cells have been typically recruited toward TAA by monoclonal antibodies eliciting antibody-dependent cellular cytotoxicity (ADCC) that engage FcγRIII/CD16A through their Fc portions, a novel class of bispecific NK cell engagers aims at the retargeting of NK cells by additionally engaging NK cell-activating receptors such as NKp46 or NKG2D [1,9,10,11].

Antibodies usually target intact cell-surface-expressed proteins that mostly lack true tumor specificity and hence are prone to mediate on-target/off-tumor effects against healthy tissues. The antigen-specific membrane-bound T cell receptor (TCR), in contrast, recognizes surface-exposed MHC-I-restricted peptides derived from a large variety of processed intracellular proteins, including oncofetal proteins and proteins harboring tumor-specific mutations, hence greatly widening the target space and offering potentially tumor-specific effector cell activation. Recognition by T cells of MHC-I (pMHC-I) complexes loaded with tumor-derived peptides is the basis of T cell-based cancer immunotherapies that require the adoptive transfer of expanded tumor-infiltrating lymphocytes or of TCR-engineered T cells, or alternatively, vaccination with mRNA or synthetic peptides [12,13,14,15].

Because the production of patient-tailored cellular products is technically demanding and difficult to standardize, it has long been attempted to extend the concept of immune cell-activating bispecific antibodies to the ectodomains of membrane-bound T cell receptors. Difficulties in the production of stable soluble TCR molecules and the naturally low affinity of TCR–pMHC-I interactions have hampered the development of soluble TCR-based agents, and only a limited number of such reagents are available so far [16]. The only clinically approved TCR-based bispecific T cell engager is the ‘immune-mobilizing monoclonal TCR against cancer’ (ImmTAC) molecule tebentafusp, specific for the gp100_280–288_ peptide presented by HLA-A*02:01 [17,18]. Another ImmTAC recognizing a peptide derived from the cancer testis antigen PRAME has been preclinically validated [19]. ImmTAC molecules are composed of a monomeric, high-affinity-matured TCR fused to a CD3ε-specific single-chain variable fragment (scFv) and have been shown to induce potent target-specific immune responses toward tumor target cells expressing <50 pMHC-I complexes with preferential activation of CD8^+^ T cells [18,19]. Recently, a MAGE-A4/MAGE-A8 peptide/HLA-A*02:01 targeted TCR-based T cell engager in the heterodimeric, Fc-conjugated TCER (T cell engaging receptor) format has been evaluated with promising results in a phase I basket study involving several solid tumor indications including head and neck cancer, melanoma and non-small cell lung cancer [20]. TCER molecules are Fc-conjugated heterodimers that combine the variable domains of a T cell receptor with the variable domains of a CD3ε-binding antibody [20,21].

Since TCR-based engagers that recruit NK cells have not been systematically studied so far, here we engineered different variants of NK cell engagers that were based on a bivalent IgG1-like structure [22], containing disulfide-stabilized ectodomains of full-length TCR beta and alpha chains combined with an augmented IgG1-hinge/Fc portion in order to harness the capacity of inducing NK cell-mediated ADCC. In novel formats of TCR-scFv-Fc fusion proteins incorporating scFv antibodies binding to activating NK cell receptors, NKp46 or CD16A, the NK cell-stimulating capacity was further potentiated. This was inspired by previously reported trifunctional NK cell-engaging anti-CD20 antibodies containing an ADCC-enhanced Fc portion and an anti-NKp46 cross-MAb [9]. Furthermore, the homodimeric TCR-scFv-Fc architecture was applied to T cell redirection using an anti-CD3ε scFv and a silenced Fc portion. For a panel of model TCRs and respective pMHC-I complexes, we demonstrate that TCR-scFv-Fc proteins possess significant potential for the activation of freshly isolated NK and T cells from peripheral blood and in vitro redirection toward tumor target cells, provided that peptide/MHC-I complexes are present in sufficient numbers.

## 2. Materials and Methods

### 2.1. TCR-Fc Fusion Protein Cloning

TCR RA14 recognizing the HCMV pp65 (495–503) peptide NLVPMVATV restricted by HLA-A*02:01 has been functionally and structurally analyzed [23] (PDB|3GSN). In addition to the wild-type RA14 sequence, the affinity-matured TCR RA14 α2.β8 developed by Wagner et al., 2019 [22], harboring the mutations TRAV-CDR3 (T108Y, Q115H) and TRBV-CDR3 (P108L, I113V, G115L), was used.

The TCR IMCgp100 recognizing the gp100/PMEL (280–288) peptide YLEPGPVTA restricted by HLA-A*02:01 and its affinity-matured variant TRAV (D93S, L96M, V97Q)/TRBV (Q50W, I51A, V52Q, N53G, I95W, G97A) have been described [18,24,25,26] (PDB|7PHR_A, PDB|7PHR_B).

The TCR 1G4 recognizing the NY-ESO-1 (157–165) peptide SLLMWITQC restricted by HLA-A*02:01 and its affinity-matured variant TRAV (T95L, S96Y) [27,28], and the TCR DMF5 recognizing the MART-1/Melan-A (26–35) peptide EAAGIGILTV and its affinity-matured variant TRBV (T54A) have been described [27,29].

Synthetic cDNAs were generated containing TRAV sequences of the TCRs mentioned above, preceded by the human TRAV24 ER leader sequence (GenBank QYZ99853, 1–22) and the human TRAC sequence (GenBank AAH63432) truncated after L99, with substitutions T48C, N33Q and N67Q [22]. Following TRAC-L99, an *Nhe*I site (coding for AlaSer) was added for scFv cloning, followed by the hIgG1 hinge-CH2-CH3 sequence. TRBV/TRBC2 sequences were cloned following a human IL-2 leader sequence (GenBank AAB86861, 1–10). The human TRBC2 sequence (GenBank AAA82687) with substitutions S57C, N70Q and C75S was either truncated after C131 by insertion of a stop codon, or a *Bcu*I site (coding for ThrSer) was added after TRBC2-F133 (coding for ThrSer) for scFv sequence insertion, respectively.

The bivalent IgG-like TCR-Fc fusion proteins consist of two TCR monomers fused to an Fc fragment of human IgG1 (hinge-CH2-CH3, GenBank BC078670, E216–K447). TCR Vβ/Cβ chains replaced VL/CL, and TCR Vα/Cα chains replaced VH/CH1 of IgG1. The TRAV/TRAC-hinge-CH2-CH3 and TRBV/TRBC polypeptide chains were joined in one open reading frame by a T2A ribosomal skipping sequence (GenBank XGT88491, 313–333). TCRα and TCRβ pairing was facilitated by the formation of two inter-polypeptide disulfide bonds, one between two artificially introduced cysteines in the constant domain (TRAC-T48C and TRBC-S57C) [30] and one between Cys95 and Cys131 in the stalk domains of TCR-Cα and -Cβ, respectively, while Cys220 in the hIgG1 hinge region was mutated to serine to avoid aberrant disulfides. IgG1 Cys251/Cys254 within the hinge domain form two intermolecular disulfide bonds, resulting in the formation of a bivalent TCR-Fc homodimer.

TCR-scFv-Fc fusion proteins additionally incorporated different scFvs C-terminal of TCRα or TCRβ: Anti-NKp46, clone NKp46-1 (VH, PDB:6IAP_H; VL, PDB:6IAP_L) [9], anti-CD16A, clone 4-LS21 (VH, GenBank AME94873.1; VL, GenBank AME94866.1) [31], anti-CD3ε, clone OKT3 (VH, GenBank A22261.1; VL, GenBank A22259.1) [32], anti-CD3ε, humanized clone UCHT1 from bsAb r3M (VL-linker-VH scFv, GenBank AJ853735) joined after a short linker sequence (Gly_3_Ser or SerGly_4_Ser). VH and VL sequences were joined using a flexible (Gly_4_Ser)_3_ linker. The OKT3 scFv was engineered in VH-linker-VL (“HGL”) and VL-linker-VH (“LGH”) orientations.

The Fc fragment of the TCR fusion proteins either contained different mutations for enhanced FcγRIII receptor binding (S239D, A330L, I332E) [33], or had a mutated *N*-glycosylation acceptor site (N297Q) for strongly reduced FcγRIII receptor binding [34,35]. All the constructs were cloned into the expression vector pcDNA3.1 (–) (ThermoFisher/Invitrogen, Waltham, MA, USA) with a hIg-κ endoplasmic reticulum (ER) signal peptide *N*-terminal to TCRα-hinge-CH2-CH3 and a Strep-Tag II C-terminal to the Fc part, and the human IL-2 ER signal peptide *N*-terminal to TCRβ. A control bsAb combining an anti-CD19 scFv (clone BCE19 [36]) with the anti-CD3ε OKT3_HGL_ scFv was engineered into the (scFv-hIgG1-scFv)_2_ format as previously described [37,38].

### 2.2. Cloning of Soluble and Membrane-Bound MHC-I Single-Chain Trimers

Peptide–MHC complexes were generated as disulfide-trapped single-chain trimers (dtSCT) either for membrane-bound or soluble expression, which consist of a peptide ligand followed by a flexible glycine-serine-rich linker, human β2-microglobulin (β2m), a second flexible linker, and the HLA-A*02:01 ectodomain, either with transmembrane/intracytoplasmic domains for membrane-bound expression or without for soluble expression. For soluble expression the MHC-I ectodomain was fused to the hinge-CH2-CH3 of mIgG2a followed by a Strep-Tag II, generating a bivalent pMHC-I dtSCT-Fc fusion protein as described in detail previously [39]. The disulfide trap in dtSCT is formed between the MHC-I α1-domain mutation Tyr84 to Cys and Cys at position 2 of the first flexible linker [39,40,41]. Membrane-bound pMHC-I-mSCT incorporated an AviTag in the linker between β_2_m and the MHC-I ectodomain and were cloned in a bicistronic construct with a T2A ribosomal skipping sequence, with an ER-targeted *E. coli* birA biotin ligase sequence appended by the ER retention motif KDEL [40]. Constructs were cloned into pcDNA3.1 (–). In gp100_280–288_ and NY-ESO-1_157–165_ dtSCTs, the C-terminal amino acids of the peptide sequences SLLMWITQC and YLEPGPVTA, respectively, were exchanged to valine to provide an improved position 9 anchor for HLA-A*02:01. In survivin_96–104_ dtSCT, Thr97 in peptide LTLGEFLKL was replaced by Met to provide an improved position 2 anchor.

### 2.3. Production of TCR-Fc Fusion Proteins and BsAbs

For recombinant protein production by Chinese hamster ovary cells (CHO-S; ThermoFisher/Invitrogen), the cells were transfected to achieve transient gene expression as previously described [42,43,44]. For maintenance culture, CHO-S cells were cultivated shaking (50 mm diameter, 100 rpm) at 37 °C and 8% CO_2_ in 100 mL complete PowerCHO-2CD medium (Lonza, Basel, Switzerland) supplemented with 8 mM GlutaMax™ (ThermoFisher/Gibco, Waltham, MA, USA), 1× HT Supplement (Merck/Sigma-Aldrich; Darmstadt, Germany) and 0.5× Antibiotic Antimycotic (Sigma-Aldrich) in 500 mL glass bottles (round bottom) until they reached 4 × 10^6^ cells/mL. For transfection, 3 × 10^8^ cells were resuspended in 100 mL complete ProCHO-4 (Lonza) supplemented with 4 mM GlutaMax™, 1× HT supplement + 0.5× Antibiotic Antimycotic. Two bottles with each 100 mL culture volume were prepared for each production batch, and 2.5 µg polyethyleneimine (PEI; Polysciences Europe GmbH, Warrington, PA, USA) and 0.625 µg plasmid DNA were added per 10^6^ cells. Following 6 h of shaking (50 mm diameter, 100 rpm) at 37 °C and 8% CO_2_, the culture was supplemented with 1 mM valproic acid (Sigma-Aldrich). The protein-rich cell culture supernatant was finally collected after 6 days of shaking (50 mm, 100 rpm) at 32 °C and 5% CO_2_ and applied to Strep-Tactin-based purification. A 0.7 cm diameter column was filled with 1.5 mL Strep-Tactin^®^ Superflow^®^ high-capacity resin and washed with PBS. Proteins were eluted using PBS supplemented with 5 mM desthiobiotin (IBA Lifesciences, Göttingen, Germany) and dialyzed against PBS. Successful purification was confirmed using SDS-PAGE.

### 2.4. Cell Lines and Culture Conditions

MCF-7 (ATCC HTB-22) and T2 (ATCC CRL-1992) were cultured in RPMI-1640 (ThermoFisher/Gibco) supplemented with 10% heat-inactivated fetal calf serum (FCS; Gibco); 2 mM GlutaMax™ and 1% Penicillin/Streptomycin (Sigma-Aldrich). MeWo (CLS Cell Lines Service, Heidelberg, Germany), SK-MEL-5 (CLS), SK-MEL-37 (Merck/Millipore, Darmstadt, Germany) and A375 (CLS) were cultured in DMEM (Gibco), supplemented with 10% heat-inactivated FCS; 2 mM GlutaMax™ and 1% penicillin/streptomycin. For subculture, adherent cells were harvested after trypsin treatment (0.05% trypsin/EDTA; Gibco).

### 2.5. NK and T Cell Isolation

Human T and NK cells from the lympho-monocytic fraction of 3–4 buffy coats from healthy donors were freshly isolated for each experiment. Buffy coats were not pooled. A negative magnetic bead-based selection was performed according to the manufacturer’s protocol using the human pan-T cell and NK cell isolation kits from Miltenyi Biotec (Bergisch Gladbach, Germany). NK and T cells were purified to 90–95% purity as determined by staining for CD56^+^/CD16^+^/CD3^−^/CD19^−^/CD14^−^ and CD3^+^/CD56^−^/CD19^−^/CD14^−^ lymphocyte populations (see Section 2.6). While healthy donors for NK cell purification were unselected, HLA-A2-negative donors were identified using mAb BB7.2 for the isolation of T cells using the HLA-A2-reactive mAb BB7.2 in flow cytometry.

### 2.6. Flow Cytometry

For discrimination of living and dead cells, flow cytometry samples were stained with the Zombie Aqua^TM^ Fixable Viability Kit (BioLegend, San Diego, CA, USA) as instructed by the manufacturer’s protocol. If required, an FcR blocking solution was applied afterwards (Human TruStain FcX^TM^, BioLegend), resuspended in PBS supplemented with 1% FCS. For staining of surface markers, the cells were collected by centrifugation and resuspended in 50 µL antibody solution (diluted in PBS with 1% FCS). To analyze TCR binding of TCR-Fc fusion proteins, the cells were resuspended in 1 µg/mL fusion protein (if not stated otherwise) diluted in 50 µL FACS buffer and incubated for 1 h at 4 °C. Afterwards, the cells were washed with FACS buffer twice. For detection of the TCR-Fc fusion protein, the cells were incubated with a PE-conjugated goat-anti-human-IgG1 secondary antibody (Jackson ImmunoResearch, West Grove, PA, USA; 50 µL per sample diluted 1:200 in FACS buffer) for 15 min at 4 °C. Before analysis, all flow cytometry samples were washed twice and fixed by resuspension in PBS supplemented with 1% FCS and 2.5% paraformaldehyde.

To measure degranulation and intracellular cytokine levels of effector cells, 4 h co-cultures were set up in the presence of GolgiPlug™ solution (1:1000; contains brefeldin A) and GolgiStop™ solution (1:1000; contains monensin) (BD Biosciences, Franklin Lakes, NJ, USA) and 0.5 µL AlexaFluor 647-conjugated anti-CD107a antibody per well (H4A3, BioLegend).

Following co-cultures for 4 or 24 h (see Section 2.8) using NK or T cells, the samples were processed for acquisition by flow cytometry. To enable the identification of effector cell populations the samples were co-stained for lineage markers using fluorochrome labeled antibodies from BioLegend: CD56 (HCD56, FITC), CD3 (UCHT1, BV510, dump channel), CD14 (M5E2, BV510), CD19 (HIB19, BV510) for NK cells, and CD3 (HIT3a, APC/Cy7), CD4 (RPA-T4, AF488), CD8 (SK1, PacificBlue), CD14 (M5E2, BV510, dump channel), CD19 (HIB19, BV510), and CD56 (HCD56, BV510) for T cells. Following fixation and permeabilization using the BD Cytofix/CytopermTM Fixation/Permeabilization Kit (BD Biosciences), NK or T cells were stained with antibodies for human interferon (IFN)-γ (4S.B3, PE-labeled, BioLegend) and tumor necrosis factor (TNF)-α (Mab11, PE-Cy7, BioLegend). For activation marker profiling, NK cells were co-stained with antibodies against CD69 (FN50, AF647) and 4-1BB/CD137 (4B4-1, PE), and T cells were co-stained for CD69 (FN50, BV421), CD25 (BC96, AF647), CD137 (CD8^+^ T cells; 4B4-1, PE), CD134 (CD4^+^ T cells; Ber-ACT45, PE/Cy7).

Anti-CD19 (HIB19, PE, BioLegend) and anti-CD20 (2H7, APC, BioLegend) were used for T2 cell surface staining. Anti-CD326 (EpCAM, 9C4, APC, BioLegend), anti-EGFR (AY13, APC, BioLegend) and anti-HER2 (24D2, PE, BioLegend) were used for MCF-7 surface staining.

### 2.7. Enzyme-Linked Immunosorbent Assay (ELISA)

To analyze pMHC-I binding of the recombinant TCR-Fc fusion proteins, 5 µg/mL pMHC-I SCT-Fc fusion protein was coated (100 µL/well diluted in PBS) onto a 96-well MaxiSorp ELISA plate (Thermo Fisher Scientific) overnight at 4 °C. Afterwards, blocking buffer (PBS supplemented with 2.5% BSA) was added for 45 min at 37 °C. Next, the TCR-Fc fusion proteins diluted in PBS/0.5% BSA were applied for 1.5 h at 37 °C in titrated concentrations. A peroxidase-conjugated anti-human IgG-Fc (Sigma-Aldrich) antibody was used for detection. Following 30 min incubation at 37 °C, TMB (3,3′,5,5′-tetramethyl-[1,1′-biphenyl]-4,4′-diamine) peroxidase substrate solution (BioLegend) was applied according to the manufacturer’s instructions, and 1 M H_2_SO_4_ was used to stop the enzymatic reaction. Absorbance of the reaction product was measured at 420 nM wavelength with 540 nm as the reference.

### 2.8. In Vitro Co-Culture of Tumor Cells with NK and T Cells

In vitro co-culture assays were used to analyze whether the recombinant TCR-Fc and TCR-scFv-Fc fusion proteins were able to induce target-directed cytotoxicity by NK cells or T cells and effector cell activation. Target cells were seeded in a 96-well plate approximately one day before co-culture (flat-bottom plate for adherent cells, round-bottom plate for suspension cells) in their respective culture medium to reach 50.000 cells/100 μL/well on the day of co-culture. If required, synthetic peptides were added to the cell suspension during seeding to reach 100 μM final concentration if not stated otherwise. After the peptide pulse, NK target cells were washed once using 200 µL of complete RPMI medium. Following overnight incubation at 37 °C and 5% CO_2_, the effector cells (E:T for NK cells 2.5:1, E:T for T cells 5:1), soluble TCR-Fc and TCR-scFv-Fc fusion proteins, and mono- and bispecific antibodies were added, diluted in complete RPMI medium, and further incubated at 37 °C and 5% CO_2_ as required. The co-culture with naïve NK cells isolated from buffy coats was analyzed after 4 h for target-directed cytotoxicity by LDH release assay, degranulation (CD107a) and intracellular cytokine levels (IFN-γ, TNF-α), and after 24 h for cell surface activation markers (CD69, CD25, 4-1BB). The co-culture with naïve T cells isolated from buffy coats was analyzed after 24 h incubation to analyze target-directed cytotoxicity, degranulation, intracellular cytokine levels and cell surface activation markers, and after 72 h to measure T cell proliferation.

### 2.9. Cytotoxicity Assay

Cytotoxicity was analyzed after 4 h incubation for NK cell co-cultures and after 24 h for T cell co-cultures by measuring lactate dehydrogenase (LDH) released by dying cells using the CyQUANT™ LDH Cytotoxicity Assay kit (Roche, Basel, Switzerland). As a positive control, 10 μL lysis buffer/well was added to tumor target cells when setting up the co-culture. To determine basal LDH levels resulting from spontaneous lysis, controls with tumor cells only or effector cells only were included. Percentage of lysis was calculated as follows:

(1) $$\%Cytotoxicity=\frac{sample\;release-effector\;cell\;spontaneous\;release-target\;cell\;spontaneous\;release}{target\;cell\;maximum\;release-target\;cell\;spontaneous\;release}\times \;100$$

### 2.10. T Cell Proliferation Assay

To measure T cell proliferation, the T cells were labeled with 1 µM CellTrace™ Violet (CTV, Thermo Fisher Scientific) according to the manufacturer’s instructions shortly before setting up the co-culture. Following 5 days of co-culture, the cells were processed for flow cytometry and stained for CD3 (HIT3a, APC/Cy7), CD4 (RPA-T4, PE) and CD8 (RPA-T8, APC).

### 2.11. Three-Dimensional Spheroid Model

To generate spheroids, 50.000 MCF-7 cells were seeded per well of a Nunclon™ Sphera™ 96-well plate in ice-cold spheroid medium (100 µL/well, DMEM/F-12 (Thermo Fisher Scientific) supplemented with 10% FCS, 2% B-27 (Thermo Fisher Scientific) and 2% Geltrex™ (Thermo Fisher Scientific) and centrifuged at 120 rpm and 4 °C for 5 min. Following 2 days of incubation at 37 °C, effector cells and active compounds were added using DMEM/F12 medium supplemented with 10% FCS and 2% B-27. After addition of NK or T cells, the co-culture was extended for 24 h for all readouts, including cytotoxicity, degranulation and intracellular cytokines, as well as activation marker profiling. To analyze the proliferation of T cells, the co-culture was extended to 5 days.

### 2.12. Generation of pMHC-mSCT/CD80 Transfectants

Human CD80 cDNA (GenBank BC042665) was cloned into the expression vector pcDNA6-V5/HisA (ThermoFisher/Invitrogen). MCF-7 cells were sequentially transfected with CD80 and membrane-bound, in vivo biotinylated pMHC-mSCT using lipofectamine™ 3000 (Thermo Fisher Scientific) and according to the manufacturer’s instructions. To generate stable cell lines, the cells were selected with 8 µg/mL blasticidin following CD80 transfection and with 600 µg/mL G418 following pMHC-SCT transfection for two weeks. To enrich for CD80-expressing cells, the cells were stained with anti-CD80 antibody (2D10, APC, BioLegend), and the stained cells were isolated with anti-APC microbeads (Miltenyi Biotec). To enrich mSCT-expressing cells following each transfection and selection process, the cells were stained with streptavidin–PE (BioLegend) (1 µL/0.5 × 10^6^ cells/100 µL) and isolated with anti-PE microbeads (Miltenyi Biotec) according to the manufacturer’s protocol.

### 2.13. Statistical Analysis

Statistical analyses were performed using GraphPad Prism v10.6.1. EC_50_ values from dose–response curves were computed using a three-parameter log(agonist) vs. response nonlinear regression model (sigmoidal curve fit) using means of response data ± s.e.m. from independent experiments with response values from 2 to 4 donors as input. The repeated-measures (RM) one-way ANOVA test with Geisser–Greenhouse correction was used to compare individual donor responses among treatment groups. Dunnett’s multiple comparisons test was applied to compare each treatment group with the non-treated (no TCR) control. Data are presented as mean values ± s.e.m. A biological replicate unit comprised all conditions (no TCR control plus various TCR-(scFv)-Fc formats) in one individual experiment. A new set of NK cell or T cell donors was used for each individual experiment. The number of donors is stated in the respective figure legends, with *p* values < 0.05 considered statistically significant (ns, *p* > 0.05; *, *p* < 0.05; **, *p* < 0.01; ***, *p* < 0.001).

## 3. Results

### 3.1. pMHC-I Binding of TCR-Fc Fusion Proteins

**Figure 1:**
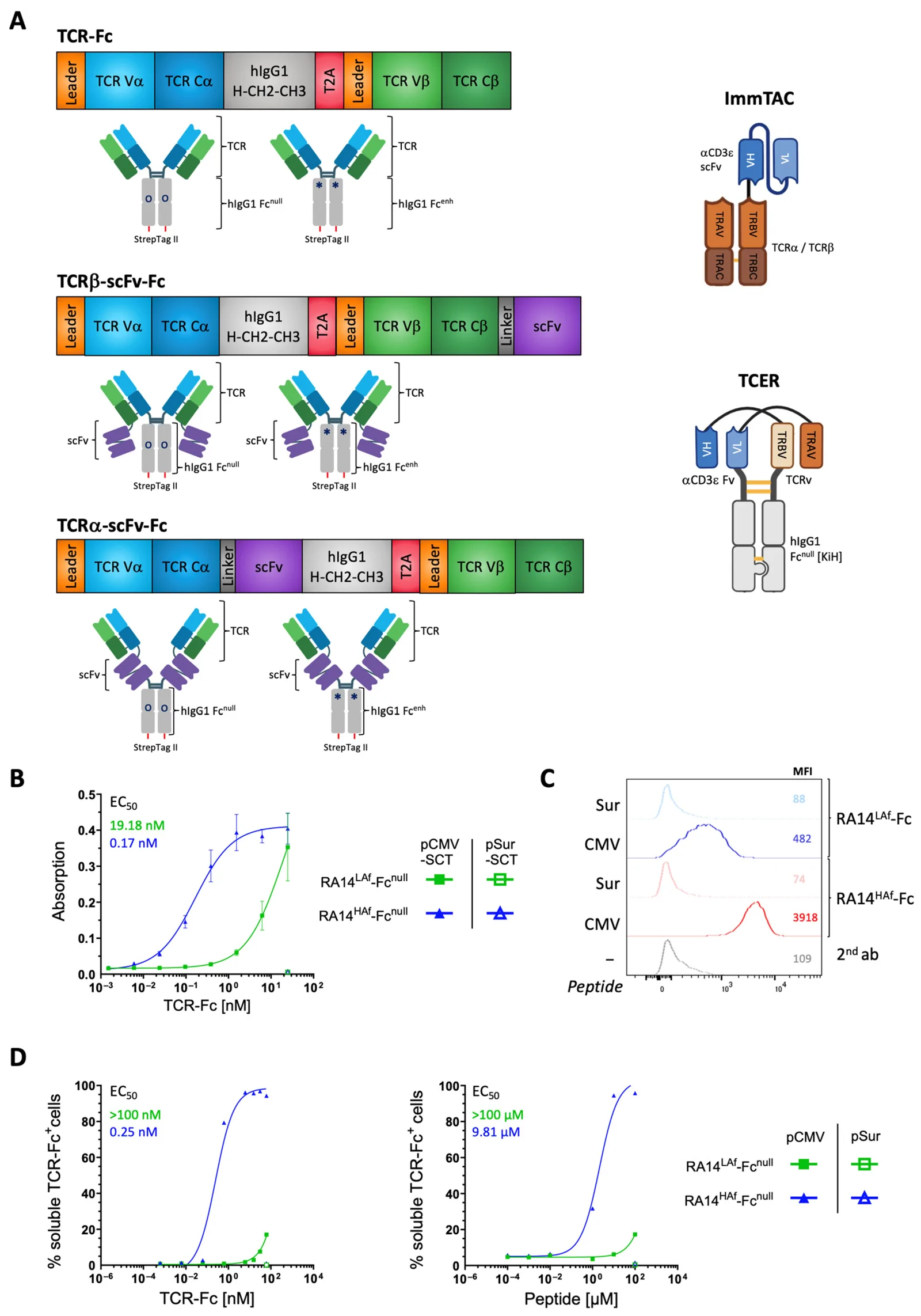
Target binding of low- and high-affinity CMV pp65-specific TCR-Fc fusion proteins. (**A**) Schematic linear representation of soluble TCR-Fc and TCR-scFv-Fc fusion proteins engineered in an IgG-like format with the TCRα chain fused to the hinge (H)-CH2-CH3 domain of human IgG1 and the TCRβ chain expressed as a separate polypeptide after a T2A sequence. TCR-scFv-Fc fusion proteins are equipped with an scFv incorporated between the C-terminus of TCRα and the hinge (TCRα-scFv-Fc) or at the C-terminus of TCRβ (TCRβ-scFv-Fc), which can be anti-NKp46 or anti-CD16A in NK cell engagers, or anti-CD3ε in T cell engagers. Fc portions can either contain mutations that abrogate FcγRIII binding (Fc^null^, “o” denotes the N297Q aglycan mutation; used in T cell engagers) or enhance it (Fc^enh^, “*” denotes mutations S239D/A330L/I332E; used in NK cell engagers). For comparison, the structures of established TCR-based ImmTAC [17,18] and TCER [20,21] T cell engagers are schematically shown on the right side. (**B**) Target binding was analyzed for TCR-Fc fusion proteins of the wild-type (low affinity, LAf) and affinity-matured (high affinity, HAf) variants of the CMV pp65-specific TCR RA14 toward plate-bound CMV pp65 (pCMV-MHC) and survivin (pSur-MHC) peptide-tethered HLA-A*02:01-Fc fusion (dtSCT-Fc) proteins by ELISA. Concentration of the TCR-Fc protein was titrated. Binding was detected using peroxidase-conjugated anti-human IgG-Fc. Shown are mean values ± s.e.m. from three independent experiments conducted in duplicate. (**C**) Representative flow cytometry staining of RA14^LAf/HAf^-Fc fusion proteins to cognate CMV pp65 (CMV) and control survivin (Sur) peptide-pulsed HLA-A2^+^ T2 cells. Binding was detected by flow cytometry using PE-conjugated anti-human IgG. (**D**) Left graph, binding of titrated amounts of RA14^LAf/HAf^-Fc to T2 cells pulsed with 100 µM of CMV pp65 (pCMV) or survivin peptide (pSur); right graph, binding of 6 nM RA14^LAf/HAf^-Fc to T2 cells pulsed with titrated amounts of pCMV peptide or 100 µM pSur for control. Binding was detected by flow cytometry using PE-conjugated anti-human IgG.

To engineer TCR-Fc-based agents, a published bivalent chimeric IgG1-like format [22] was adopted with modifications (Figure 1A). VL/CL of hIgG1 were replaced by TCR Vβ/Cβ domains, and VH/CH1 of hIgG1 were replaced by TCR Vα/Cα domains, respectively, followed by the hinge and Fc fragment (CH2-CH3). TCRα-hinge-CH2-CH3 and TCRβ were combined in a single open reading frame using a T2A ribosomal skipping sequence [45]. Efficient TCRα and TCRβ chain pairing was promoted by an artificially introduced disulfide bond (Cα:T48C and Cβ:S57C) [30] as well as a natural disulfide bridge in the stalk domains of TCR-Cα and TCR-Cβ. Strep-Tactin-based purification of assembled TCR-Fc fusion proteins was enabled by a Strep-tag II incorporated at the C-terminus of the Fc part. NK- and T-cell-reactive scFv antibodies were incorporated either between TCR-Cα and Fc (TCRα-scFv-Fc) or at the C-terminus of TCR-Cβ (TCRβ-scFv-Fc) after a short flexible linker. In TCR-based NK cell engagers, an enhanced hIgG1-Fc part was used, whereas an aglycan Fc^null^ was incorporated in TCR-based T cell engagers and in control constructs. The different molecular architectures of two TCR-based T cell engagers in clinical use (ImmTAC, TCER) are schematically shown for comparison in Figure 1A.

We first compared, in TCR-Fc constructs, the wild-type and affinity-matured versions [22] of TCR RA14 [23], specific for HLA-A*02:01/HCMV pp65(495–503). Following successful production in CHO-S cells and purification to >95% purity (Supplemental Figure S1), target binding of TCR-Fc fusion proteins was analyzed for sensitivity and specificity using ELISA (Figure 1B) and flow cytometry (Figure 1C). A soluble CMV-pp65 peptide-tethered HLA-A*02:01 SCT-mIgG2a-Fc fusion protein (pCMV-SCT) was adsorbed to an ELISA plate, and binding of TCR-hIgG1-Fc fusion proteins was detected using an hIgG-specific peroxidase-conjugated antibody. Survivin peptide-tethered HLA-A*02:01 SCT-Fc (pSur-SCT) served as a specificity control. The TCR-Fc titration showed EC_50_ values of 19 nM for the wild-type RA14^LAf^-Fc and 0.17 nM for the affinity-matured RA14^HAf^-Fc.

For the analysis by flow cytometry, HLA-A2^+^ TAP-deficient lymphoblastoid T2 cells were pre-incubated with pp65_495–503_ peptide NLVPMVATV, or survivin_96–104(T97M)_ peptide LMLGEFLKL for control, to replace endogenous cell surface-displayed HLA-A2 peptide ligands. Figure 1C shows a representative staining of peptide-loaded T2 cells (100 µM) with 6 nM RA14^LAf^-Fc or RA14^HAf^-Fc fusion proteins. Control peptide-pulsed T2 cells were not stained above background levels of untreated T2 cells. To analyze functional avidities, TCR-Fc and peptide concentrations were titrated. The binding strength was clearly dependent on TCR affinity; for the RA14^HAf^-Fc, an EC_50_ value of 0.25 nM was measured, while the RA14^LAf^-Fc variant did not reach saturation at 100 nM TCR-Fc. The peptide titration revealed an EC_50_ value of 9.8 µM peptide for RA14^HAf^-Fc, while the binding of RA14^LAf^-Fc remained at 20% of the maximal RA14^HAf^-Fc binding at the highest usable peptide concentration (100 µM).

### 3.2. NK Cell Engagement by TCR-Fc^enh^ Fusion Proteins

**Figure 2:**
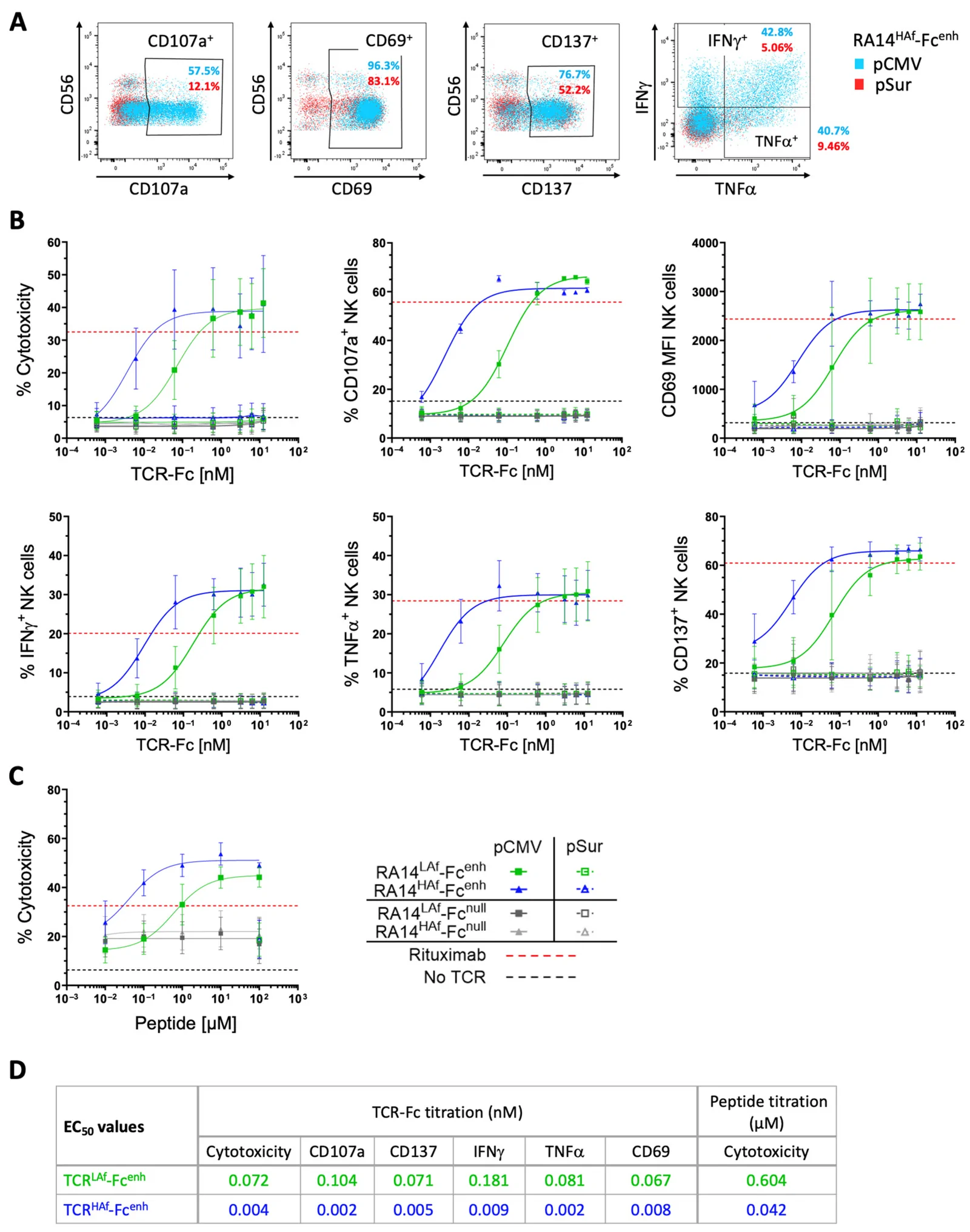
NK cell activation and cytotoxicity against peptide-pulsed T2 cells in the presence of fusion proteins of CMV pp65-specific TCR RA14 high-affinity (HAf) and RA14 low-affinity (HAf) variants containing an ADCC-enhanced Fc part (Fc^enh^) or a silenced Fc part (Fc^null^) for control. (**A**) Degranulation and activation of CD56^+^ NK cells were analyzed using flow cytometry by staining for CD107a, CD69, CD137, and intracellular IFNγ and TNFα after co-culture with T2 cells pulsed with CMV pp65 (CMV) peptide (blue) or survivin (Sur) peptide (red). (**B**) Titration of RA14^HAf/LAf^ TCR-Fc fusion protein in co-cultures of freshly isolated peripheral blood NK cells with T2 cells pulsed with either pp65 peptide (pCMV, solid lines) or survivin peptide (pSur, dashed lines) for control. Either 6 nM ADCC-competent anti-CD20 mAb rituximab (red dashed line) or a co-culture without TCR (blue dashed line) was included for comparison. Cytotoxicity was evaluated using an LDH release assay. NK cell degranulation and activation were analyzed using flow cytometry by staining for CD107a, CD69, CD137, and intracellular staining for IFNγ or TNFα. Mean values ± s.e.m. from independent experiments are shown (cytotoxicity, CD107, CD69, IFNγ, TNFα, all *n* = 3; CD137, *n* = 2). (**C**) Titration of pp65 peptide concentration in co-cultures of peptide-pulsed T2 cells with NK cells in the presence of CMV pp65-specific TCR-Fc^enh/null^ fusion proteins used at 6 nM concentration. The control peptide pSur was used at 100 µM only. Cytotoxicity was evaluated following 4 h co-culture using an LDH release assay. Additional read-out assays of the peptide titration are shown in Supplementary Figure S2. (**D**) EC_50_ values for mean response curves computed using a non-linear regression model are displayed.

NK cell redirection was facilitated by using a mutated Fc part (Fc^enh^) to enhance binding to the ADCC-inducing FcγRIIIa/CD16A receptor, by incorporating scFvs recognizing either CD16A (clone 4-LS21) [31] or the activating natural cytotoxicity receptor NKp46 (clone NKp46-1) [9], or by a combination of Fc^enh^ and scFv. NK cell redirection of the high- and low-affinity pp65/HLA-A2 TCR-Fc^enh/null^ constructs was evaluated in co-culture experiments with peptide-pulsed T2 cells by analyzing target cell killing, NK cell activation and degranulation (Figure 2). The activation of freshly isolated NK cells was assessed by flow cytometry staining for intracellular IFNγ and TNFα, as well as the activation markers CD69 and CD137. Because NK cells showed a high baseline expression of CD69 (Figure 2A), in the following experiments we show stimulation-dependent differences in CD69 median fluorescence intensity (MFI) values. In the presence of the high-affinity TCR-Fc^enh^ fusion protein, the co-culture with CMV pp65 peptide-pulsed T2 cells resulted in increased NK cell degranulation and activation compared to the co-culture with survivin peptide-pulsed cells, as indicated by increased expression of CD107a, CD69, CD137, and high intracellular levels of IFNγ and TNFα in mostly CD56^dim^ NK cells from buffy coat PBMC (Figure 2A).

To analyze the relevance of TCR affinity and antigen density for NK cell redirection using soluble TCR-Fc constructs, concentrations of TCR-Fc^enh^ fusion proteins (Figure 2B, T2 cells preloaded with 100 µM pp65 peptide) and CMV pp65 peptide were titrated (Figure 2C, Supplementary Figure S2A, 6 nM TCR-Fc). The ADCC-inducing, anti-CD20 mAb rituximab was included for comparison (red dashed lines). The CD20 antigen is expressed at low to intermediate levels by T2 cells (Supplementary Figure S3E). Survivin peptide-pulsed T2 cells and the TCR-Fc^null^ constructs served as negative controls. RA14 TCR-Fc^enh^ fusion proteins increased pp65 peptide-specific NK cell-mediated cytotoxicity, NK cell degranulation and NK cell activation in a TCR-Fc- and peptide concentration-dependent manner. No response was observed toward survivin peptide-pulsed T2 cells or using the TCR-Fc^null^ control constructs, demonstrating that the observed effects were antigen-specific and Fc receptor-dependent. Saturating NK responses were reached with ~6 nM TCR-Fc, which was used for the following NK cell co-cultures. EC_50_ values of the affinity-matured RA14^HAf^-Fc^enh^ ranged from 2 to 8 pM and were thus 8 to 52 times lower than those of the wild-type RA14^LAf^-Fc^enh^ variant (Figure 2D). Likewise, 6- to 40-fold differences regarding the functional responses induced by TCR RA14^HAf/LAf^-Fc^enh^ constructs were observed for the titration of pulsed peptide (Figure 2D, Supplementary Figure S2B). Notably, NK cell activation and cytotoxicity values obtained with 6 nM rituximab corresponded to only 5–50 pM RA14^HAf^-Fc^enh^ (Figure 2B). Furthermore, levels of cytotoxicity and cytokine induction obtained with 6 nM rituximab corresponded to NK cell responses in the presence of 6 nM RA14^HAf^-Fc^enh^ after T2 loading with 20–200 nM peptide, which is 500–5000-fold lower than maximum peptide densities (Figure 2C, Supplementary Figure S2A). We conclude that in the presence of sufficient quantities of pMHC-I complexes, NK cell activation by high-affinity TCR-Fc^enh^ constructs can be similar to or exceed the NK cell activation effected by an ADCC-mediating monoclonal antibody against a weakly expressed tumor antigen.

### 3.3. NK Cell Redirection Toward Tumor Cells by TCR-scFv-Fc^enh^ Fusion Proteins

**Figure 3:**
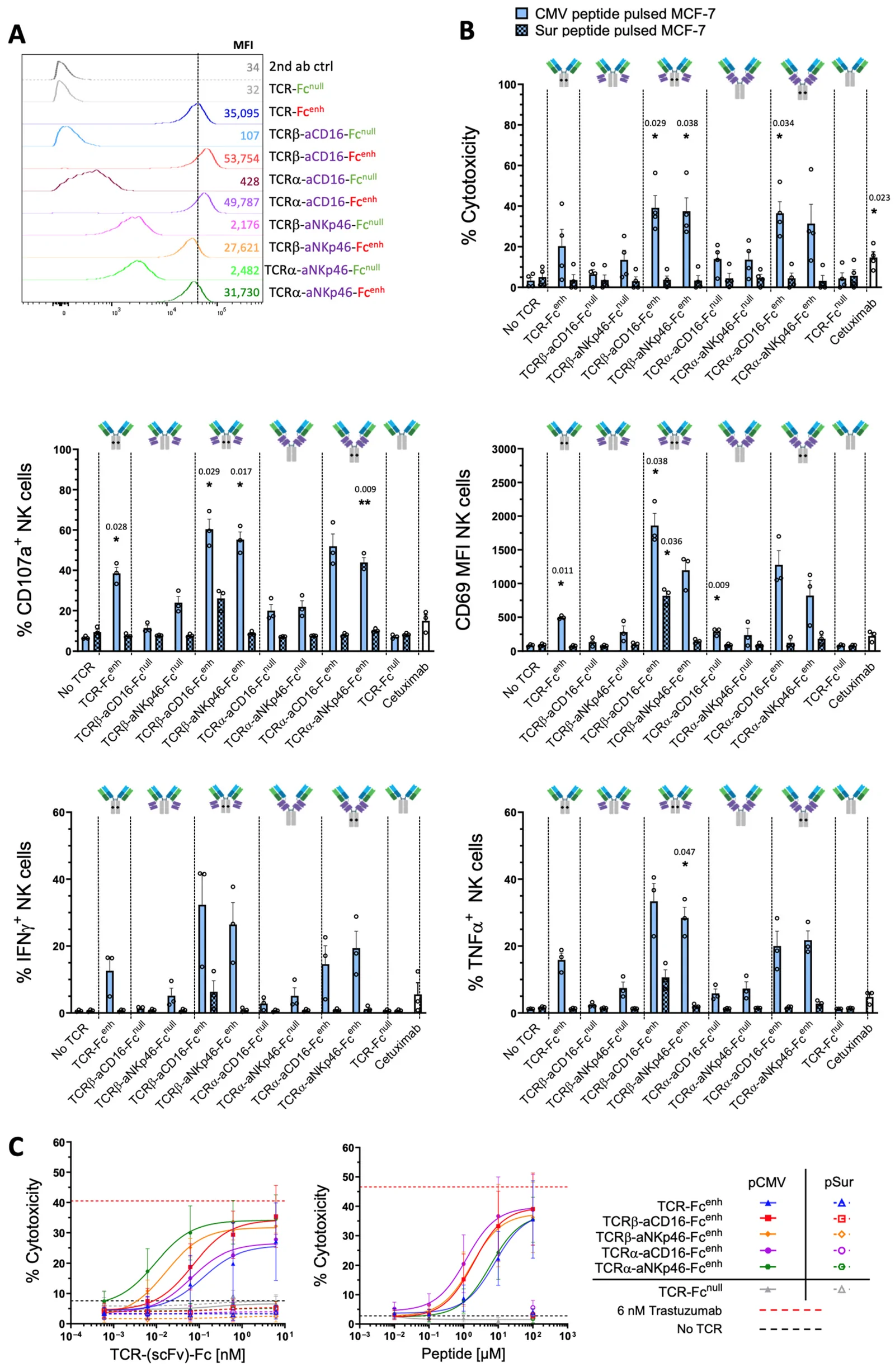
Comparison of different CMV pp65-specific high-affinity TCR-Fc^enh/null^ and TCR-scFv-Fc^enh/null^ fusion proteins used for NK cell redirection. (**A**) NK cell binding was analyzed by flow cytometry using 30 nM of the indicated TCR-Fc^enh/null^ and TCR-scFv-Fc^enh/null^ fusion proteins in which scFv antibodies recognizing CD16A or NKp46, respectively, were fused at the C-terminus of the TCRβ chain (“TCRβ-aCD16/aNKp46-Fc^enh/null^”), or inserted between the C-terminus of the TCRα chain and the hIgG1-Fc^enh^ or the Fc^null^ part (“TCRα-aCD16/aNKp46-Fc^enh/null^”). Mean fluorescence intensities of staining with TCR-scFv-Fc^enh/null^ plus anti-human-IgG1-PE are indicated. (**B**) Functional evaluation of the indicated TCR-Fc^enh/null^ and TCR-scFv-Fc^enh/null^ fusion proteins with HLA-A2^+^ MCF-7 breast cancer cells as targets. MCF-7 cells were preincubated with 100 µM pCMV or pSur and co-cultured with NK cells in the presence of 6 nM RA14^HAf^ TCR-Fc^enh/null^ and TCR-scFv-Fc^enh/null^ fusion proteins. An amount of 6 nM of the ADCC-inducing anti-EGFR mAb cetuximab was applied for comparison. Cytotoxicity was analyzed using an LDH release assay. NK cell degranulation and activation were analyzed using flow cytometry by staining for CD107a, CD69, intracellular IFNγ and TNFα. Mean values ± s.e.m. and data points from 3 experiments using *n* = 3 different NK cell donors are shown. Significance compared to “No TCR” was evaluated using a repeated-measures one-way ANOVA test for CMV pp65 and survivin peptide-pulsed targets separately for each donor, followed by Dunnett’s multiple comparison test. Significant *p* values are indicated as numbers; *, *p* < 0.05; **, *p* < 0.01. (**C**) Left panel: NK cytotoxicity assay with MCF-7 cells preincubated with 100 µM pCMV (solid lines) or pSur (dashed lines) in the presence of titrated concentrations of the indicated RA14^HAf^ TCR-Fc^enh^ and TCR-scFv-Fc^enh^ fusion proteins. Omission of the TCR fusion protein and RA14^HAf^ TCR-Fc^null^ served as negative controls. An amount of 6 nM of the ADCC-inducing anti-HER2 mAb trastuzumab was included for comparison. LDH release was measured to analyze cytotoxicity after 4 h co-culture. Mean values ± s.e.m. from 3 experiments using *n* = 3 NK cell donors are shown. (**C**) Right panel: MCF-7 cells were preincubated with titrated concentrations of pCMV, while the pSur control peptide was used at 100 µM only. The indicated TCR-Fc^enh^ and TCR-scFv-Fc^enh^ fusion proteins, and trastuzumab for comparison, were added at 6 nM concentration. Mean values ± s.e.m. from 3 independent experiments using *n* = 3 NK cell donors are shown. EC_50_ values for cytotoxicity assays with titrated TCR-[scFv]-Fc^enh^ fusion proteins and titrated pCMV corresponding to (**C**) as obtained from nonlinear regression analysis of mean values are shown in Table 1. NK cell degranulation and activation by titrated TCR-[scFv]-Fc^enh^ fusion proteins and titrated CMV peptide are shown in Supplementary Figure S4.

Next, we studied the function of NK cell-activating scFv antibodies incorporated in TCR-Fc constructs either C-terminally to the TRBC domain or the TRAC domain, respectively, of the affinity-matured pp65/A2-reactive TCR (see Figure 1A and schematic drawings in Figure 3B). Binding of TCR-scFv-Fc molecules to NK cells was analyzed by flow cytometry (Figure 3A). While for the TCR-Fc^null^ fusion protein no binding to NK cells could be detected, the TCR-Fc^enh^ fusion protein showed strong binding, which was not significantly improved by the anti-CD16A scFv nor the anti-NKp46 scFv, suggesting that the binding of Fc^enh^ to FcγRIII clearly dominated. Using TCR-scFv-Fc^null^ constructs, a weak Fc-independent NK cell staining by the anti-CD16A scFv and a moderate Fc-independent staining by the anti-NKp46 scFv was detected. NK cell staining by fusion proteins containing anti-CD16A scFv or NKp46 scFv, respectively, was slightly superior in the TCRα-scFv-Fc^null^ as compared with the TCRβ-scFv-Fc^null^ format.

The capacity to elicit NK cell cytotoxicity against cancer cells, activation and effector cytokine secretion of TCR-Fc^enh/null^ and TCR-scFv-Fc^enh/null^ fusion proteins was analyzed in parallel using peripheral blood NK cells and pp65 peptide-pulsed HLA-A*02:01^+^ MCF-7 breast cancer cells as targets in 4 h co-culture assays (Figure 3B). Of note, staining of MCF-7 cells with an anti-HLA-A2 antibody showed ~4-fold lower HLA-A2 expression levels on the cell surface (Supplementary Figure S3A) and were stained ~8-fold less efficiently by the RA14^HAf^ TCR-scFv-Fc^null^ construct after pulsing MCF-7 or T2 cells with 100 µM CMV pp65 peptide (Supplementary Figure S3B). For comparison, we included the anti-EGFR antibody cetuximab, which elicits NK cell cytotoxicity via ADCC, at the same 6 nM concentration as the TCR-(scFv)-Fc fusion proteins. EGFR is weakly expressed by MCF-7 target cells (Supplementary Figure S3F and [38]).

TCR-Fc^null^ fusion proteins induced no NK cell responses above background defined by the omission of a TCR fusion protein, demonstrating the efficacy of Fc silencing. For the TCRα-NKp46/CD16A-scFv-Fc^null^ and the TCRβ-NKp46-scFv-Fc^null^ constructs, we noted tendencies to increase LDH release, NK cell degranulation, CD69 upregulation and cytokine release above levels of TCR-Fc^null^ and no-TCR controls, which is consistent with the weak to moderate NK cell binding of TCR-scFv-Fc^null^ constructs. Despite its efficient NK cell binding by flow cytometry (Figure 3A), the TCR-Fc^enh^ fusion protein showed only intermediate capacity to elicit NK cell activation, which was significant only for CD107a upregulation (Figure 3B). Incorporation of either the anti-CD16A or the anti-NKp46 scFv into TCR-Fc^enh^ fusion proteins resulted in significantly enhanced induction, or clear tendencies below significance, of CMV peptide-specific cytotoxicity, degranulation, and activation, as well as cytokine secretion, with a preference for the scFv positioned C-terminally of the TCRβ chain, suggesting an additive effect of agonistic antibodies targeting NKp46 or CD16A in short-term NK co-cultures.

To investigate the potency of TCR-scFv-Fc constructs for antigen-specific NK cell activation, we titrated the concentration of TCR-scFv-Fc constructs in NK cell–MCF-7 co-cultures as well as the concentration of CMV peptide used for the exogenous loading of MCF-7 cell-surface expressed HLA-A2 molecules (Figure 3C and Table 1). With EC_50_ values of 17 and 9 pM, respectively, the TCRβ-NKp46-scFv-Fc^enh^ and TCRα-NKp46-scFv-Fc^enh^ fusion proteins showed the lowest EC_50_ values for eliciting cytotoxicity, which were 7–13-fold lower than the EC_50_ value of scFv-free TCR-Fc^enh^ (119 pM). Comparable results were obtained when analyzing NK degranulation, activation and cytokine secretion in the presence of titrated amounts of TCR-(scFv)-Fc fusion proteins (Supplementary Figure S4A,C). EC_50_ values for peptide titrations in the presence of a fixed TCR-(scFv)-Fc concentration (6 nM) showed similar differences in the range of 5–23-fold between the potencies of TCR-Fc^enh^ and the most sensitive TCR-scFv-Fc^enh^ fusion proteins, TCRβ-αNKp46-Fc^enh^ or TCRα-αCD16-Fc^enh^ (Supplementary Figure S4B,C).

Notably, the TCRβ-CD16A-scFv-Fc^enh^ fusion protein caused partial NK cell activation by control peptide-pulsed MCF-7 cells (Figure 3B) and elicited peptide-independent NK cell activation in TCR-scFv-Fc^enh^ titrations (Supplementary Figure S4B), suggesting that positioning of the anti-CD16A scFv at the C-terminal end of the TCRβ chain caused FcγRIII cross-linking on NK cells independently of the interaction between pHLA-A2 and TCR. Therefore, this fusion protein was excluded from further investigation. Except for trastuzumab eliciting a superior kill of MCF-7 targets than any of TCR-scFv-Fc^enh^ constructs (Figure 3C), NK cell activation by TCRα/β-NKp46-scFv-Fc^enh^ fusion proteins in terms of CD107 externalization, CD69, or 4-1BB induction, as well as IFNγ or TNFα expression (Supplementary Figure S4), were comparable or even slightly higher than NK responses induced by an equimolar concentration (6 nM) of the ADCC-mediating antibody trastuzumab recognizing the HER2 receptor that is strongly expressed by MCF-7 cells (Supplementary Figure S3F and [38]).

### 3.4. T Cell Redirection Toward Tumor Cells by TCR-scFv-Fc^enh^ Fusion Proteins

After establishing the novel TCR-scFv-Fc format for the recruitment of NK cells toward pMHC-I complexes, we sought to apply the same RA14^HAf/LAf^ TCR-based model system to the recruitment of T cells. Because T cell activation via CD3ε has been successfully employed in several bispecific antibodies developed by others and us [1,2,37,38] and in ImmTAC TCR-based T cell engagers [18], we decided to use an anti-CD3ε scFv in homodimeric TCR-scFv-Fc molecules.

**Figure 4:**
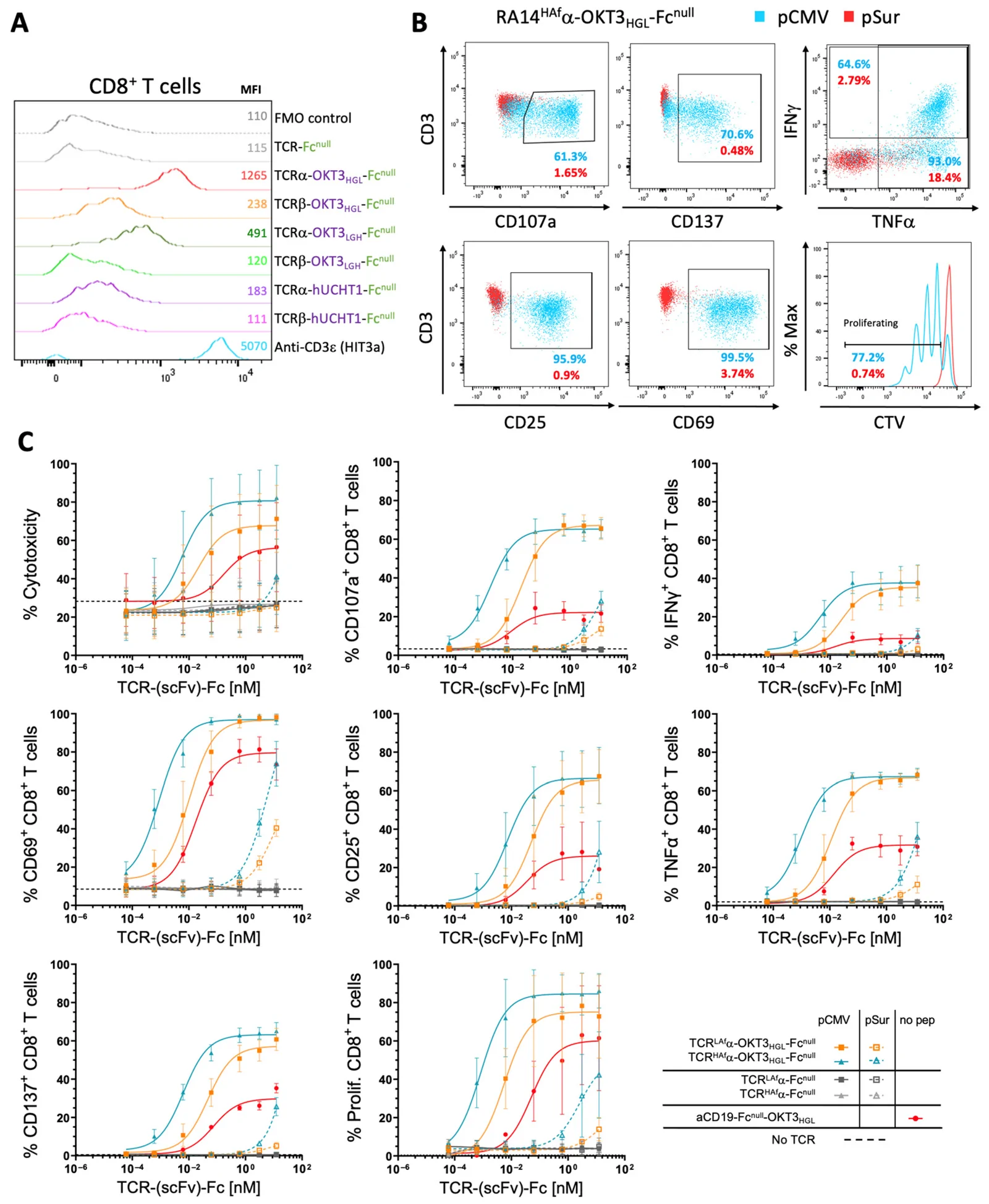
T cell activation by TCR-scFv-Fc^null^ fusion proteins. (**A**) Binding of 6 different variants of TCR RA14^LAf^ anti-CD3ε-Fc^null^ fusion proteins to CD8^+^ T cells was analyzed by flow cytometry using 6 nM fusion protein for staining and anti-human-IgG1-PE. Fusion proteins contained the CMV pp65-specific TCR RA14^HAf^ and OKT3 scFv antibodies in either V_H_-glycine-serine linker-V_L_ (HGL) or V_L_-linker-V_H_ (LGH) format or humanized UCHT1 scFv in LGH format, conjugated at the C-terminal end of either TCRα or TCRβ. RA14^HAf^-Fc^null^ was included as a negative control, and a PE-conjugated anti-CD3ε (clone HIT3a) antibody was included as a positive control. Mean fluorescence intensities of a representative staining are shown. FMO, “fluorescence-minus-one” control staining without PE-conjugated secondary antibody. (**B**) T cell activation after co-culture of unactivated CD3^+^ T cells from PBMC with peptide-pulsed T2 cells in the presence of the RA14^HAf^α-OKT3_HGL_-Fc^null^ T cell engager. Cytotoxicity was analyzed following 24 h co-culture using an LDH release assay (*n* = 3). T cell degranulation and activation were analyzed by flow cytometry following a 24 h co-culture of CD3^+^ T cells with T2 cells pulsed with 100 µM pCMV, or pSur for control, and by staining for CD69 (*n* = 4), CD107a (*n* = 4), CD25 (*n* = 3), CD137 (*n* = 4), and intracellular IFNγ (*n* = 4) and TNFα (*n* = 4). CD3 staining of CD8^+^ T cells is displayed. Proliferation of CD3^+^/CD8^+^ T cells stimulated with pCMV- or pSur-treated T2 cells was analyzed by cell division-dependent dilution of CTV in CTV-labeled T cells after 5 days of co-culture (*n* = 2). (**C**) Titration of TCR RA14^HAf/LAf^α-OKT3_HGL_ scFv-Fc^null^ fusion proteins in co-cultures of CD3^+^ T cells with T2 cells loaded with 100 µM CMV pp65 peptide (pCMV, solid lines), or survivin peptide (pSur, dashed lines) as specificity control. As controls for non-specific T cell activation, RA14^HAf/LAf^α-Fc^null^ constructs without anti-CD3ε scFv were used, or T cell engagers were omitted (“No TCR”). A homodimeric anti-CD19(BCE19)-scFv-hIgG1-Fc^null^-anti-CD3ε(OKT3_HGL_)-scFv bsAb was titrated for comparison. Mean values ± s.e.m. from 3 experiments using 3 different T cell donors are shown. EC_50_ values for T cell activation and cytotoxicity assays with titrated TCR RA14^HAf/LAf^α-OKT3_HGL_ scFv-Fc^null^ fusion proteins and anti-CD19-hIgG1-Fc^null^-anti-CD3ε bsAb are shown in Table 2. Results from peptide titration experiments are shown in Supplementary Figure S5A.

For redirection of T cells, the TCR-Fc constructs contained an Fc part with a mutated *N*-glycosylation site to prevent FcγRIIIa binding (Fc^null^). ScFv antibodies derived from the established anti-CD3ε mAb OKT3 in V_L_-linker-V_H_ (OKT3_LGH_) or V_H_-linker-V_L_ (OKT3_HGL_) orientations, or a scFv derived from anti-CD3ε mAb UCHT1, respectively, were tested for their efficacy to bind and activate CD3^+^ T cells after cloning the scFv C-terminally to the TCR α-chain or β-chain of the CMV pp65-specific wild-type TCR RA14. The six recombinant RA14^LAf^ TCR-scFv-Fc molecules were produced in CHO-S cells, purified via StrepTactin (Supplementary Figure S1), and tested by flow cytometry for binding to naïve T cells freshly isolated from buffy coats using anti-hIgG-PE (Figure 4A). TCR-Fc^null^ fusion protein without anti-CD3ε scFv did not detectably bind to T cells. The OKT3 scFv in V_H_-linker-V_L_ format inserted between the TCRα chain and the IgG1 hinge domain produced the strongest staining of CD3^+^ T cells (Figure 4A) and was therefore chosen for further experiments. Fusion proteins with the anti-CD3ε scFv cloned C-terminally to TCRβ generally labeled T cells with weaker intensity than counterparts with the anti-CD3ε scFv inserted between TCRα and hinge-Fc.

To demonstrate the functionality of TCR-anti-CD3ε-Fc fusion proteins, T2 cells pulsed with CMV pp65 peptide, or survivin peptide for control, were co-cultured with freshly isolated, unseparated CD3^+^ T cells for 24 h in the presence of 0.6 nM RA14^Haf^ TCRα-OKT3_HGL_-scFv-Fc^null^ fusion proteins and analyzed by flow cytometry for the induction of activation markers, degranulation and cytokine secretion (Figure 4B). We detected a profound and peptide antigen-specific activation of CD3^+^/CD8^+^ T cells in terms of upregulation of CD69, CD25, and CD137(4-1BB); early and late activation markers; upregulation of CD107a indicating externalization of cytotoxic granules; and a profound induction of IFNγ/TNFα double-positive CD3^+^/CD8^+^ T cells. A similar activation was observed for CD3^+^/CD4^+^ T cells, which expectedly showed only a minor degree of CD107a upregulation. The proliferation of CD3^+^/CD8^+^ T cells was measured after 5 days. A proliferating fraction of 77% demonstrated sustained activation. TCR and peptide titration experiments with purified CD3^+^ T cells and T2 target cells were conducted to study the potency and specificity of CMV pp65/A2-reactive TCR-OKT3_HGL_-scFv-Fc fusion proteins in detail. Using the LDH release assay and flow cytometry, we observed 3.5- to 11-fold differences between RA14^HAf^ and RA14^LAf^ TCRα-OKT3_HGL_-scFv-Fc^null^ proteins in EC_50_ values regarding cytotoxicity against peptide-loaded T2 cells and CD107a externalization; upregulation of activation markers CD69, CD25, and CD137; proliferation; and intracellular IFNγ or TNFα expression by CD8^+^ T cells (Figure 4C, Table 2). Depending on the read-out, RA14^HAf^ TCRα-OKT3_HGL_-scFv-Fc^null^ concentrations required to achieve half-maximal T cell activation ranged between 1 and 7 pM (Table 2), demonstrating the excellent sensitivity of our TCR-based T cell engagers. Similar results were obtained when analyzing activated CD3^+^/CD4^+^ T cells for CD69, CD25, CD134(OX40), IFNγ, and TNFα upregulation and proliferation.

RA14 TCRα-OKT3_HGL_-scFv-Fc^null^ fusion proteins did not exhibit the same degree of antigen specificity as compared with RA14-based NK cell engagers since RA14^HAf/LAf^ TCRα-OKT3_HGL_-scFv-Fc^null^ fusion proteins began to elicit T cell activation at concentrations ≥ 0.63 nM when T2 target cells were pulsed with the control peptide. This effect, which was most conspicuous for the early activation marker CD69, required the presence of the OKT3_HGL_ scFv (Figure 4C). When the sensitivities of RA14^HAf^ and RA14^LAf^ TCRα-OKT3_HGL_-scFv-Fc^null^ fusion proteins (60 pM input) were compared in peptide titration experiments analyzed for T cell cytotoxicity, degranulation, activation and proliferation, we observed 130- to 188-fold differences in EC_50_ values depending on the assay used (Supplementary Figure S5A,B). Hence, TCRα-OKT3_HGL_-scFv-Fc^null^ fusion proteins appear to sensitively monitor pMHC-I densities that are expected to influence differential dwelling times of TCRs with high vs. low affinity.

To compare the efficacy of TCR-anti-CD3ε-scFv-Fc^null^ molecules with a similarly formatted bispecific antibody, a homodimeric tetravalent anti-CD19-scFv-hIgG1-Fc^null^-anti- CD3ε(OKT3_HGL_)-scFv bsAb was engineered and titrated in parallel with TCR-anti-CD3ε-Fc fusion proteins (Figure 4C; Table 2). CD19 is strongly expressed by T2 cells (Supplementary Figure S3E). Unexpectedly, the anti-CD19 x anti-CD3ε bsAb performed inferior to even the RA14^LAf^ TCRα-OKT3_HGL_-scFv-Fc^null^ fusion protein when comparing equimolar input quantities. Hence, similar to NK cell-engaging RA14^HAf^ TCR-scFv-Fc proteins mentioned above, the efficacy of T cell-engaging RA14^HAf^ TCR-scFv-Fc proteins can be equal to or even superior to bispecific antibody-based T cell engagers depending on antigen density and antibody affinity.

### 3.5. T Cell Activation by TCR-scFv-Fc^enh^ Fusion Proteins Is Supported by Costimulation

TCR affinity and density of peptide–MHC-I complexes on the target cell are important factors influencing the potency of soluble TCR-based bispecific agents. In line with the lower expression of endogenous HLA-A2 (Supplementary Figure S3A) and the likely presence of TAP-dependent peptides that are more difficult to exchange, MCF-7 cells showed a lower degree of TCR RA14^HAf^-Fc^null^ binding following a CMV pp65 peptide pulse compared to T2 cells (Supplementary Figure S3B). Accordingly, in co-cultures of T cells and MCF-7 cells (Supplementary Figure S6A), the RA14^LAf^ and RA14^HAf^ TCRα-OKT3_HGL_-scFv-Fc^null^ fusion proteins induced lower responses regarding cytotoxicity and T cell activation and had greater EC_50_ values compared to co-cultures with T2 cells (Table 2, Supplementary Figure S6D). Furthermore, MCF-7 breast cancer cells lack the costimulatory CD80 molecules expressed by B-lymphoblastoid T2 cells [46].

Simultaneous administration of a costimulatory anti-EpCAM-Fc^null^-anti-CD28 bsAb that redirects the CD28 receptor to highly cell surface-expressed EpCAM that is highly expressed by MCF-7 cells (Supplementary Figure S3F and [38]), led to a drastic improvement of the potency of the RA14^LAf^ TCRα-OKT3_HGL_-scFv-Fc^null^ fusion protein in terms of T cell activation by retargeted MCF-7 cells, and increased saturation levels of late activation markers CD25 and CD137 in the presence of RA14^HAf^ TCRα-OKT3_HGL_-scFv-Fc^null^ (Supplementary Figure S6A,C), indicating a potential benefit of TAA-targeted costimulation when using low-affinity TCR-Fc T cell engagers. An anti-EpCAM-scFv-Fc^null^-OKT3_HGL_-scFv bsAb [38] used for comparison induced T cell activation ranging between RA14^HAf^ and RA14^LAf^ OKT3_HGL_-scFv-Fc^null^ fusion proteins. As expected from our own reported results [38], the efficacy of the anti-EpCAM x anti-CD3ε bsAb was also clearly enhanced by the addition of the costimulatory anti-EpCAM x anti-CD28 bsAb (Supplementary Figure S6A,C).

**Figure 5:**
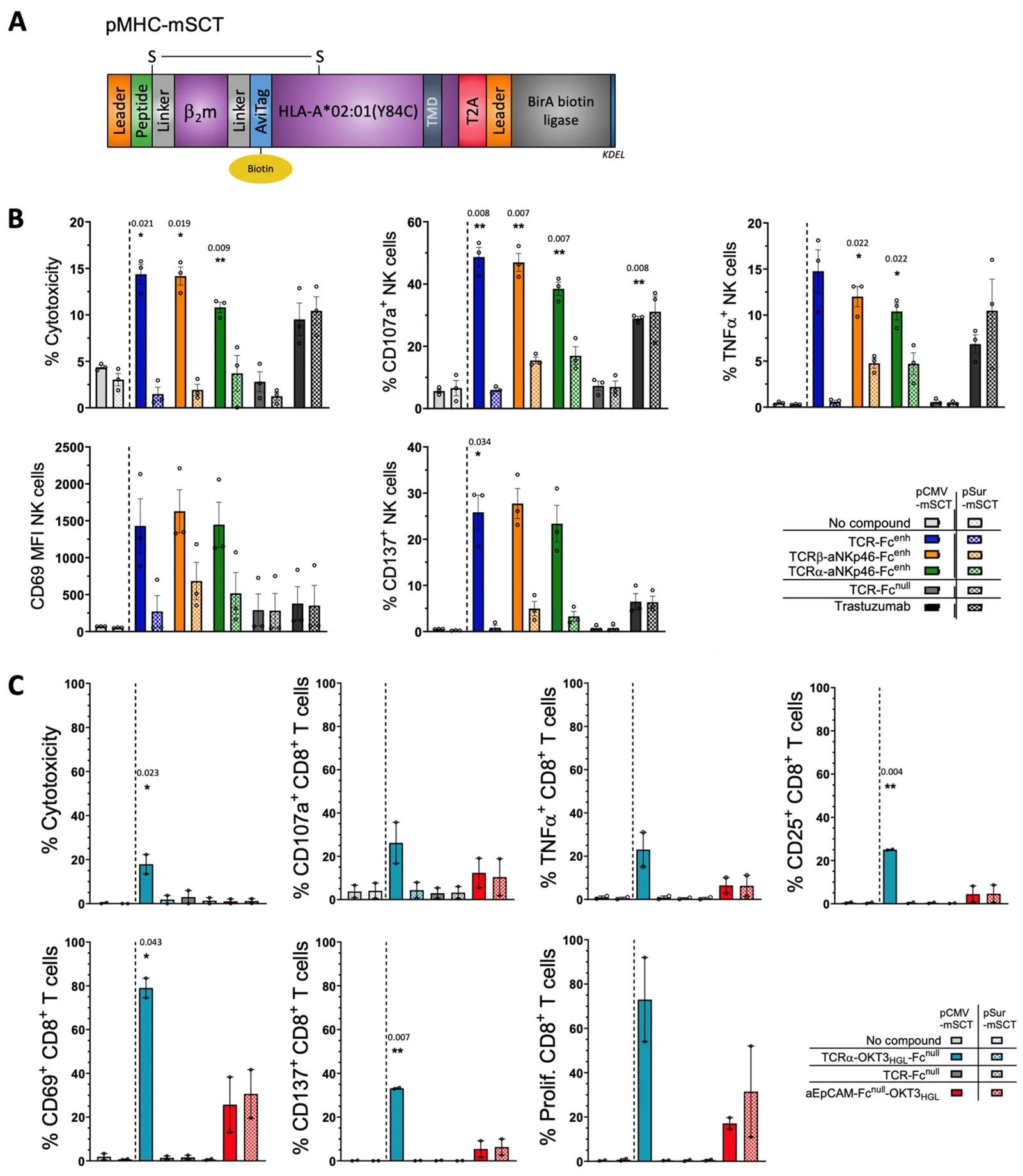
NK and T cell cytotoxicity against mSCT-transfected MCF-7 spheroids in the presence of TCR-scFv-Fc NK and T cell engager proteins. (**A**) Molecular design of biotinylated, peptide-tethered HLA-A*02:01 single-chain trimers with a transmembrane domain (pMHC-mSCT) used for transfection of MCF-7 cells. (**B**) NK cell co-culture with spheroids of MCF-7 cells transfected with mSCT containing either CMV pp65 peptide (pCMV-mSCT) or survivin peptide (pSur-mSCT) as a control, for 24 h. RA14^HAf^-Fc^enh^ or RA14^HAf^β/α-anti-NKp46 scFv-Fc^enh^ were added to co-cultures at 6 nM concentration, as indicated. Omitted TCR fusion protein and RA14^HAf^-TCR-Fc^null^ served as negative controls. ADCC-competent anti-HER2 mAb Trastuzumab (6 nM) was included for comparison. Target cell lysis was evaluated using an LDH release assay. NK cell degranulation and activation were analyzed using flow cytometry by staining for CD107a, CD69, CD137, and intracellular IFNα and TNFα. Significance compared to the co-culture without TCR was analyzed by RM one-way ANOVA test for the CMV and survivin groups separately for each of the *n* = 3 NK cell donors followed by Dunnett’s multiple comparison test. Significant *p* values are indicated as numbers; *, *p* < 0.05; **, *p* < 0.01. Individual data points and mean values from 3 independent experiments ± s.e.m. are shown. (**C**) Co-cultures of pMHC-mSCT-transfected MCF-7 spheroids with T cells in the presence of 0.6 nM RA14^HAf^ TCRα-OKT3_HGL_-Fc^null^, or RA14^HAf^ TCRα-Fc^null^ or without TCR-Fc (“No compound”) for control. Anti-EpCAM scFv-Fc^null^-anti-OKT3_HGL_ scFv bsAb (0.6 nM) was tested for comparison. Cytotoxicity was analyzed using an LDH release assay after 24 h co-culture. T cell degranulation and activation were evaluated using flow cytometry by staining for CD107a, CD25, CD69, CD137, and intracellular IFNα or TNFα after 24 h co-culture. T cell proliferation was analyzed using CTV-labeled T cells following co-culture for 5 days. Significance was analyzed by RM one-way ANOVA test for the CMV pp65 and survivin groups separately for each of *n* = 2 T cell donors compared to the “no compound” control followed by Dunnett’s multiple comparison test. Significant *p* values are indicated as numbers; *, *p* < 0.05; **, *p* < 0.01. Individual data points and mean values from 2 independent experiments ± s.e.m. are shown.

Next, we asked whether a strong increase in pMHC-I expression plus ectopic CD80 expression would rescue the comparatively weak TCR-scFc-Fc-mediated T cell responses to peptide-pulsed MCF-7 cells. To this end, the experiment was repeated using MCF-7 cells transfected with CD80 and membrane-bound CMV pp65 peptide-tethered HLA-A2 single-chain trimers (pCMV-mSCT, Figure 5A). Transmembrane SCTs were biotinylated at a biotinylation acceptor site within a linker sequence through coexpressed birA biotin ligase in order to allow for detection of transfected mSCT molecules in the presence of endogenous cell surface MHC-I molecules. Expression of the CMV pp65 peptide-tethered mSCT in a subpopulation of transfected MCF-7 cells was confirmed by staining with the CMV pp65-specific TCR-Fc construct and human Fc-specific secondary antibody (Supplementary Figure S3C), demonstrating the excellent reactivity of TCR-Fc fusion proteins with peptide-tethered MHC-I complexes. Using pCMV-mSCT/CD80-cotransfected MCF-7 cells as targets, we observed an even higher increase in the potency of RA14^LAf/HAf^ TCRα-OKT3_HGL_-scFv-Fc^null^ fusion proteins with EC_50_ values in the lower picomolar range (Supplementary Figure S6B,D). MCF-7 expressing a survivin/A2 mSCT complex served as a peptide specificity control and, similar to the results with peptide-pulsed T2 reported above (Figure 4C), elicited non-specific responses at high concentrations of RA14^LAf/HAf^ T cell engagers. Interestingly, the costimulatory effect of CD80 could still be augmented by addition of the anti-EpCAM x anti-CD28 bsAb. The latter effect was also apparent for cytotoxicity and T cell activation induced by the anti-EpCAM x anti-CD3ε bsAb (Supplementary Figure S6B,D).

### 3.6. NK and T Cell Redirection Toward Tumor Cell Spheroids by TCR-scFv-Fc^enh^ Fusion Proteins

Next, a tumor spheroid model was employed that would mimic a solid tumor more closely than a tumor cell monolayer. Because loading with exogenously added synthetic peptide was found to be unsuitable, as insufficient quantities of peptide entered the interior of spheroids by diffusion, peptide presentation was enabled by transfection of MCF-7 cells with pMHC-mSCT (Figure 5A). In 24 h co-cultures of MCF-7/pCMV-mSCT tumor cell spheroids with naïve NK cells, RA14^HAf^-TCR-Fc^enh^, RA14^HAf^α-NKp46-scFv-Fc^enh^ and RA14^HAf^β-NKp46-scFv-Fc^enh^ fusion proteins induced significant levels of antigen-specific NK cell-mediated target cell lysis, NK cell degranulation, and NK cell activation (CD137, TNFα upregulation), while there was a non-significant tendency to upregulate CD69 (Figure 5B). In 24 h co-cultures of MCF-7/pp65 mSCT tumor cell spheroids with naïve CD3^+^ T cells, 0.6 nM RA14^HAf^ TCRα-OKT3_HGL_-scFv-Fc^null^ induced target cell lysis, T cell degranulation, T cell activation, and T cell proliferation, whereas MCF-7/Sur mSCT cells were not stimulatory. The results demonstrated successful antigen-specific NK cell or T cell redirection in the spheroid model. Notably, 6 nM trastuzumab used for comparison induced lower NK cell responses than an equimolar dose of RA14^HAf^α/β-NKp46-scFv-Fc^enh^ NK cell engager, and 0.6 nM anti-EpCAM-scFv-Fc^null^-OKT3_HGL_-scFv bsAb [38] triggered lower levels of T cell activation than an equimolar dose of RA14^HAf^ TCRα-OKT3_HGL_-scFv-Fc^null^ T cell engager (Figure 5C). We conclude NK and T cell engagers in the TCR-scFv-Fc format can be effective against tumor cell spheroids provided that the pMHC-I density is sufficient.

### 3.7. NK and T Cell Engagement by TCR-scFv-Fc^enh^ Molecules Targeting pMHC-I Complexes Containing TAA Peptides

**Figure 6:**
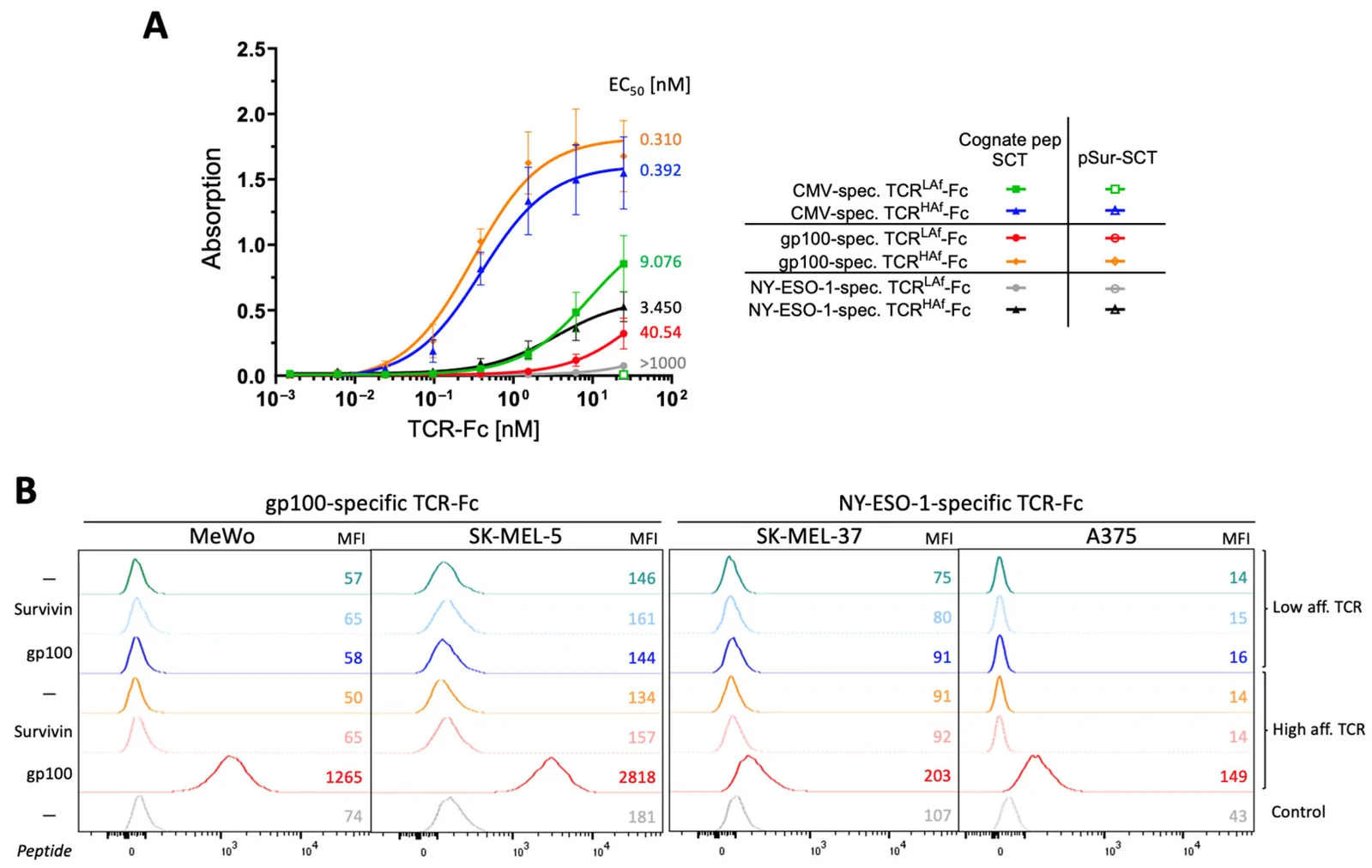
Analysis of tumor peptide-reactive TCR-Fc fusion proteins. (**A**) Binding of wildtype/low-affinity TCR-hIgG1-Fc and mutant/high-affinity TCR-Fc fusion proteins specific for HLA-A*02:01-restricted gp100 and NY-ESO-1 peptides, and CMV pp65-specific RA14 TCR^HAf/LAf^ for comparison, was analyzed by binding to plate-bound cognate dtSCT-mIgG2a-Fc, or survivin dtSCT- mIgG2a-Fc for control, by ELISA. Concentrations of the TCR-Fc proteins were titrated. Binding was detected using peroxidase-conjugated anti-human IgG-Fc. Shown are mean values ± s.e.m. from three independent experiments performed in duplicate. EC_50_ values of binding curves are indicated. (**B**) Flow cytometric analysis of melanoma cell lines MeWo, SK-MEL-5, SK-MEL-37 and A375 stained with 6 nM TCR-IMCgp100^HAf/LAf^-Fc^enh^ and TCR-1G4^HAf/LAf^-Fc^enh^ fusion proteins. Melanoma cells were left untreated or preloaded with 100 µM of cognate gp100_280–288_ or NY-ESO-1_157–165_ peptides, or survivin peptide for control.

After establishing that TCR-(scFv)-Fc^enh^ and TCR-scFv-Fc^null^ fusion proteins mediated NK and T cell redirection in the CMV pp65-reactive RA14 TCR model, we set out to engineer similar recombinant TCR fusion proteins using sequences from the HLA-A*02:01-restricted TCR 1G4 recognizing the NY-ESO-1_157–165_ peptide [27] and the HLA-A*02:01-restricted TCR IMCgp100 against the gp100_280–288_ peptide [18,47]. A wildtype TCR variant and a published affinity-matured variant were included for evaluation. For the wildtype and affinity-matured gp100-specific TCR, K_D_ affinity constants of 26 µM and 30 pM, respectively, were reported [18]. In the case of TCR 1G4, affinity maturation decreased the K_D_ from 9.3 µM to 0.73 µM [27]. All variants were producible in the IgG-like TCRα-[scFv]-Fc^enh/null^//TCRβ-[scFv] formats (Supplementary Figure S1) and showed peptide antigen-specific and concentration-dependent binding as evaluated by ELISA using immobilized SCT-Fc molecules with tethered gp100 or NY-ESO-1 peptides (Figure 6A) and by flow cytometry using peptide-pulsed T2 cells (Supplementary Figure S3G). Survivin peptide-tethered SCT-Fc served as a control for peptide specificity. EC_50_ values for binding between high- and low-affinity TCR-Fc variants of IMCgp100 and 1G4 differed by factors of 130 and >300, respectively, and thus principally confirmed enhanced TCR binding following affinity maturation while not reproducing previously reported differences between K_D_ values [18]. The binding of IMCgp100^HAf^-TCR-Fc to its cognate SCT was comparable to the binding of RA14^HAf^-TCR-Fc, which was included for comparison, with EC_50_ values of 310 pM and 392 pM, respectively, while the 1G4^HAf^-TCR-Fc fusion protein bound less efficiently (EC_50_ ~3.4 nM).

TAA-specific TCR-Fc binding was next evaluated toward tumor cell lines endogenously expressing tumor-associated antigens and HLA-A2. The cell lines used included the gp100-expressing melanoma cell lines MeWo and SK-Mel-5 and the NY-ESO-1-expressing melanoma cell lines SK-Mel-37 and A375. These cell lines had previously been used for evaluation of gp100- and NY-ESO-1/HLA-A2-specific TCRs [18,48,49,50,51]. HLA-A2 expression levels, as analyzed by flow cytometry with HLA-A2-specific antibody BB7.2, varied greatly between the cell lines SK-Mel-5, MeWo, SK-Mel-37 and A375 (Supplementary Figure S3D). TCR-Fc binding to the matched TAA-expressing tumor cell line was evaluated using flow cytometry (Figure 6B). No binding was detected toward the untreated TAA-expressing tumor cell lines using 60 nM of the low- and high-affinity TCR-Fc fusion proteins. Melanoma cells pulsed with 100 µM of the cognate TAA-derived peptides showed strong binding of the IMCgp100^HAf^ TCR-Fc and weak binding of the 1G4^HAf^ TCR-Fc, consistent with the reported differences in affinity and varying HLA-A2 expression levels, while wild-type TCR-Fc variants did not bind. Hence, endogenous expression levels of gp100/NY-ESO-1 peptide-loaded HLA-A2 complexes appeared to be below the threshold of detection for our TCR-Fc constructs.

**Figure 7:**
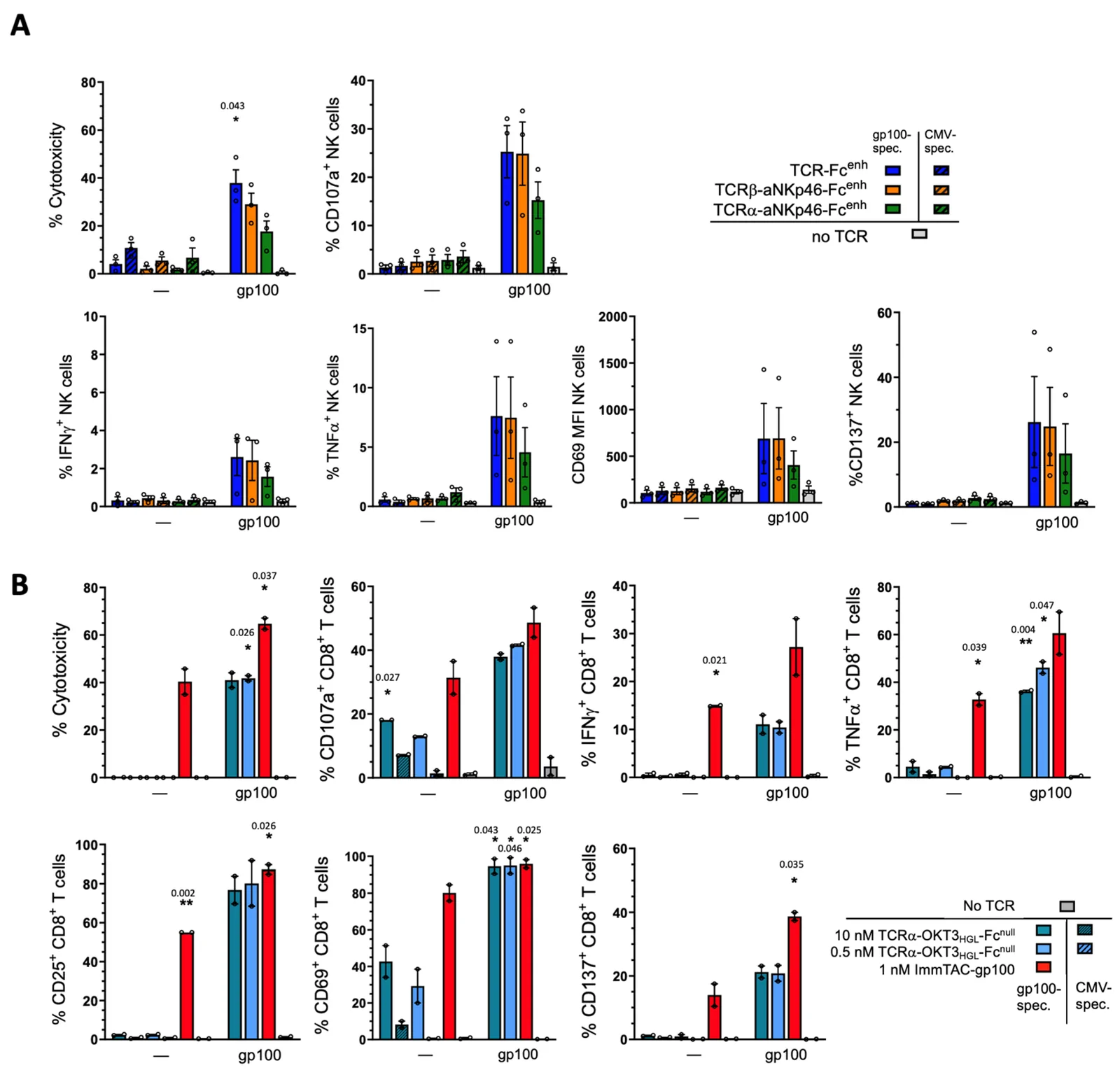
Functional analysis of tumor peptide–reactive TCR-Fc fusion proteins. (**A**) Co-culture of freshly isolated NK cells with SK-MEL-5 melanoma cells expressing endogenous gp100 in the presence of the TCR-gp100^HAf^-Fc^enh^ and TCR-IMCgp100^HAf^β/α-anti-NKp46-Fc^enh^ NK cell engagers (6 nM). CMV pp65-specific TCR-RA14^HAf^-Fc^enh^ and TCR-RA14^HAf^β/α-anti-NKp46-Fc^enh^ NK cell engagers were included as peptide-specificity controls. SK-MEL-5 cells were pulsed with 100 µM gp100 peptide or left untreated. Cytotoxicity was analyzed by LDH release assay. NK cell degranulation and activation were evaluated using flow cytometry by staining for CD107a, CD69, CD137, and intracellular IFNγ or TNFα. (**B**) Co-culture of freshly isolated T cells with SK-MEL-5 melanoma cells in the presence of 0.5 nM/10 nM TCR-IMCgp100^HAf^α-anti-CD3ε(OKT3_HGL_ scFv)-Fc^null^ or 1 nM ImmTAC-gp100 (tebentafusp). SK-MEL-5 cells were pulsed with 100 µM gp100_280–288_ peptide or left untreated. Cytotoxicity was analyzed by LDH release assay. T cell degranulation and activation were evaluated using flow cytometry. Significance compared to the co-culture without TCR was analyzed by RM one-way ANOVA test for the CMV and survivin groups separately for each of the NK cell donors (**A**; *n* = 2) and T cell donors (**B**; *n* = 3), followed by Dunnett’s multiple comparison test. Significant *p* values are indicated in numbers; *, *p* < 0.05; **, *p* < 0.01. Individual data and mean values ± s.e.m. are shown.

We next evaluated whether TAA-specific TCR-Fc constructs were nevertheless able to induce NK redirection toward target cells with naturally low pMHC-I levels. Co-cultures were set up with the affinity-matured IMCgp100 NK cell-targeting TCR-[anti-NK46-scFv]-Fc fusion proteins and the gp100-expressing SK-MEL-5 (Figure 7A). As specificity controls, respective RA14 fusion proteins were used. In the presence of 6 nM TCR-Fc^enh^, TCRβ-anti-NKp46-Fc^enh^ and TCRα-anti-NKp46-Fc^enh^ versions of TCR-IMCgp100, in 2 NK cell donors we detected tendencies toward increased cytotoxicity, NK cell degranulation and NK cell activation when SK-MEL-5 cells were loaded with exogenous gp100 peptide, while untreated SK-MEL-5 targets were not stimulatory (Figure 7A). Similar results were obtained in co-cultures of NY-ESO-1-expressing A375 cells with TCR-[anti-NK46-scFv]-Fc fusion proteins containing the affinity-matured 1G4 TCR (Supplementary Figure S6). Also in this case, NK cell cytotoxicity and activation could only be achieved after pulsing A375 cells with 100 µM synthetic NY-ESO-1 peptide.

In co-cultures of T cells and SK-MEL-5 cells, we compared TCR-IMCgp100^HAf^ TCRα-OKT3_HGL_-scFv-Fc^null^ fusion proteins side by side with the benchmark compound tebentafusp [18]. As can be inferred from published data [25], tebentafusp consists of the an anti-CD3ε scFv (humanized UCHT1) *N*-terminally linked via a Gly_4_Ser linker to the ectodomain of the TCRα chain of TCR-IMCgp100^HAf^ in association with the TCRβ chain of TCR-IMCgp100^HAf^. This structure thus clearly differs in the molecular design from [TCRα-scFv-Fc]_2_/[TCRβ]_2_ constructs used here (Figure 1A). For a fair comparison of the monovalent ImmTAC design to bivalent TCR-scFv-Fc fusion proteins, TCR-scFv-Fc were used in a two-fold lower molar concentration. In the presence of 1 nM tebentafusp, we observed an increase in cytotoxicity, T cell degranulation and T cell activation using untreated and gp100 peptide-pulsed SK-MEL-5 cells (Figure 7B). An amount of 0.5 nM gp100-specific TCRα-OKT3_HGL_-Fc^null^ elicited T cell responses only against gp100 peptide-pulsed SK-MEL-5 targets. Upon increasing the concentration to 10 nM in the co-culture with untreated tumor cells, a rather moderate but significant CD107a and CD69 upregulation was observed compared to the co-culture without TCR, while no change was observed for other analyzed parameters of T cell activation. No significant difference in T cell responses was observed between 10 and 0.5 nM gp100-specific TCRα-anti-CD3-Fc^null^ when gp100 peptide-pulsed SK-MEL-5 targets were used, suggesting that sufficient antigen density was the critical factor for an equivalent performance of TCRα-anti-CD3-Fc^null^ fusion proteins.

## 4. Discussion

MHC-restricted peptides derived from intracellular proteins are attractive targets for tumor immunotherapy as they offer recognition of a multitude of intracellular antigens that are not accessible to conventional antibodies [12]. Various cell-based approaches have been investigated for treatment, including adoptive transfer of expanded polyclonal tumor-infiltrating lymphocytes, T cell clones or TCR-engineered patient T cells [12,13,14,52]. Due to challenges in achieving standardized, efficacious and safe therapy procedures employing patient-tailored cellular products, cell-based approaches are more difficult to translate into a broadly available therapy than antibodies or antibody–drug conjugates.

This insight has stimulated the development of recombinant fusion proteins that combine the extracytoplasmic domains of TCR alpha and beta chains with agonistic mini-antibodies targeting the CD3ε subunit of T cell-expressed TCR complexes, thus enabling crosslinking of tumor cells expressing pMHC-I complexes with any CD3^+^ T cell regardless of their inherent TCR specificities. However, the often insufficient stability of recombinant TCR ectodomains and the low affinity of TCR–pMHC-I interactions imposed obstacles to the routine development of T cell-engaging soluble TCR-based reagents, and only a limited number of such reagents are under clinical investigation so far [16,53]. In an alternative strategy, TCR-mimic antibodies recognizing peptide–MHC complexes have been developed for both anti-CD3ε-based T cell engagers and antibody–drug conjugates [52,54,55]. TCR-mimic antibodies, however, tend to exhibit a lower degree of pHLA-I selectivity as compared with affinity-enhanced TCRs [56]. While TCR-based T cell engagers have been more widely studied, TCR-based recombinant molecules that recruit NK cells toward tumors have not been systematically analyzed. Two earlier studies reported the ADCC-mediating function of p53/HLA-A2-specific [57] and of γδ TCR hIgG1-Fc fusion proteins [58], respectively.

In this study, we developed a new format of soluble TCR-based bispecific engagers for the redirection of NK cells, for which a published bivalent IgG-like format [22] was modified and improved. In sTCR-Fc fusion proteins, two disulfide-stabilized TCR monomers were fused to the hinge and Fc parts of human IgG1. This bivalent design is expected to yield a higher avidity toward pMHC-I complexes compared to a monomeric format, as suggested by the superior avidity of soluble TCR-based tetramers [53] (Low et al., 2012) or dextramers [59]. To optimally harness the highly effective activating NK cell receptor CD16A/FcγRIIIa [10,11,60], which is able to elicit ADCC independently of coactivating receptors, we introduced ADCC-enhancing mutations into the Fc portion of human IgG1 [33]. Since NK cells purified from peripheral blood poorly respond to single activation receptors [61,62], we sought to enhance the function of our homodimeric sTCR-Fc fusion proteins by inclusion of agonistic scFv antibodies targeting NKp46 [9] or CD16A [31], respectively.

For the systematic evaluation of different formats of TCR-scFv-Fc-based NK engagers, the HCMV pp65_495–503_ peptide-specific TCR RA14 was used [22,23]. To assess the importance of TCR affinity for a soluble TCR-based approach, the wildtype variant with an equilibrium *K_D_* of 1.48 µM was compared to an affinity-enhanced variant of 62 nM, as previously determined in the bivalent TCR-Fc format by surface plasmon resonance [22]. In this study, soluble IgG-like RA14^LAf^ and RA14^HAf^ TCRs showed a target-specific binding dependent on concentration and TCR affinity, essentially confirming previous results [22]; however, the difference in avidities between RA14^LAf^-Fc and RA14^HAf^-Fc was 4–5-fold larger when probing immobilized pMHC-I with TCR-Fc molecules (see Figure 1B), as compared with probing of immobilized TCRs with pMHC-I complexes [22]. Using RA14^HAf^-Fc as a staining tool for peptide-pulsed T2 cells, a steep response curve was observed with an EC_50_ value of ca. 41 ng peptide/mL, while RA14^LAf^-Fc binding was considerably weaker and no saturation could be achieved in the RA14^LAf^-Fc titration. This result clearly confirmed the importance of TCR affinity maturation for effective binding of soluble TCR constructs to pMHC-I-displaying cells [51,63]. Likewise, RA14^LAf^-Fc showed a strongly decreased binding to cells pulsed with low peptide concentrations compared to the high-affinity variant, which is in accordance with a previous report studying the sensitivity of differently affine, soluble gp100-specific TCR constructs [18].

Consistent with RA14^LAf/HAf^-Fc binding, a correlation between NK cell activation and TCR affinity was observed in co-cultures of pp65 peptide-pulsed target cells in the presence of RA14^LAf/HAf^ TCR-Fc^enh^ fusion proteins used for NK cell redirection. NK engager-induced responses regarding target cell lysis, NK cell degranulation and activation were dependent on TCR-Fc concentration and TCR affinity. Importantly, target cells pulsed with high concentrations of a control HLA-A2 ligand from the survivin tumor antigen were not lysed, suggesting that the peptide specificity of TCR RA14 governed NK cell cytotoxicity. While this represents a desirable outcome, it cannot be generalized, and peptide antigen specificity needs to be demonstrated for each TCR of choice. We also showed that IgG-like TCRs were not only effective in the co-culture with peptide-pulsed T2 cells expressing high levels of HLA-A2, but also for NK cell retargeting toward the breast cancer cell line MCF-7 expressing intermediate levels of HLA-A2, thus demonstrating a broader potential of TCR-Fc-based NK cell engagers.

The systematic analysis of various variants of NK cell targeting constructs containing either an anti-NKp46 scFv or an anti-CD16A scFv, incorporated after the free C-terminus of the TCR β-chain or the Fc-linked TCR α-chain, showed a correlation between the strength of NK cell binding measured by flow cytometry and the ability of the constructs to retarget NK cells. TCR-scFv-Fc^null^ constructs that mediated binding only via the NK cell-targeting scFv showed weak NK cell staining and had small NK cell-activating effects in the co-culture compared to fusion proteins harboring the enhanced glycosylated Fc part. This finding indicated that the anti-NKp46 scFv or anti-CD16A scFv moieties rather functioned in a costimulatory manner, while NK cell activation through the enhanced glycosylated hIgG1 Fc part was dominant. Thus, the combination of the enhanced Fc part and an scFv specific for NKp46 or CD16A augmented the potency of NK cell engagers concerning target cell lysis, NK cell degranulation and activation. This additive effect of multiple NK cell receptor engagement is in full agreement with a previous study evaluating anti-NKp46–Fc^enh^–anti-CD20 NK cell engagers [9], as the NK engager containing an Fc part with enhanced FcγRIIIa binding showed stronger tumor cell lysis compared to a variant with an aglycan non-FcR-binding Fc part, or compared to a CD20-specific FcR-binding antibody without a second scFv.

In titration experiments, the highest stimulatory capacities were observed for TCRβ-anti-NKp46-Fc^enh^ and TCRα-anti-NKp46-Fc^enh^ fusion proteins. Notably, NK responses to TCR-scFv-Fc fusion proteins were stronger than those induced by equimolar amounts of the ADCC-mediating mAb cetuximab binding to EGFR, which is expressed at low levels by MCF-7 cells, and comparable to those induced by the ADCC-mediating mAb trastuzumab targeting the highly expressed HER2 antigen. While TCR-scFv-Fc NK cell engagers generally showed an excellent specificity for cognate pMHC-I complexes, the TCRβ-anti-CD16A-Fc^enh^ fusion protein showed a moderate but significant peptide-unspecific response, considering NK cell activation and degranulation that was absent using the alternative TCRα-anti-CD16A-Fc^enh^ format. We speculate that greater steric availability of the TCRβ-linked anti-CD16 scFv caused an overshooting CD16 clustering or NK cell cross-linking in *trans*, thereby triggering NK cell activation without pMHC-I engagement. On a similar topic, comparing different bispecific IgG-scFv formats with scFvs positioned C- or *N*-terminally of the IgG light or heavy chains, Fc-induced ADCC activity was previously found to be dependent on scFv positioning, and fusion of the scFv C-terminally of the C_H_3 domain even led to loss of ADCC function, probably due to steric hindrance [64,65]. We conclude that not only the positioning of the scFv is critical for efficient NK cell retargeting but also the nature of the (co)activating NK cell receptor itself. Reportedly, targeting NKp46 with agonistic antibodies appears to be advantageous over NKG2D [9].

TCR-Fc^enh^ NK cell engagers incorporating the gp100- and NY-ESO-1-reactive high-affinity TCRs elicited cytotoxicity against SK-MEL-5 and A375 melanoma cell lines, respectively, only after preincubation of these target cells with cognate peptide. We conclude that our TCR-based NK cell engagers were not sensitive enough to enable activation, cytokine release and cytotoxicity by naïve peripheral blood NK cells toward low numbers of cognate peptide MHC-I complexes resulting from natural peptide processing.

Taken together, the functional efficacy of the novel TCR-scFv-Fc depended mainly on TCR affinity and the presence of an ADCC-enhanced Fc part triggering NK cells through FcγRIIIa (CD16A), while additional single-chain antibodies either mediating cross-linking of the activating NK cell receptor NKp46, or of CD16 independently of Fc binding had only additive activating effects. The finding that the novel TCR-based NK cell engager molecules lacked sufficient sensitivity to target tumor cells presenting low amounts of pMHC-I complexes derived from natural antigen processing is a limitation of this study that needs to be addressed in future experiments using larger numbers of NK cell donors to better compensate for inter-donor variabilities in ADCC function, which can be caused by the expression of higher or lower affinity FcγRIIIa variants [66,67].

Before an improved format may be suitable for (pre)clinical development, we hope to achieve functional improvements of TCR-based NK cell engagers by reducing the molecular size by taking out the constant parts of the T cell receptor alpha and beta chains while maintaining the essential ADCC-competent Fc part in fusion proteins, and furthermore by coadministration of bispecific antibodies that crosslink a TAA such as HER2 with the activating NK cell receptor such as NKG2D [66,67]. Preliminary experiments showed that inclusion of NK checkpoint inhibitor antibodies targeting the inhibitory NK cell receptors NKG2A or KIR2DL1, 2, 3, respectively, did not significantly improve the response of freshly isolated NK cells from different blood donors toward tumor targets in the presence of TCR-scFv-Fc fusion proteins, which may have been related to low levels of NKG2A and KIR receptor engagement by target cells.

In order to utilize the TCR-scFv-Fc format for T cell redirection, six different variants were analyzed in this work to first determine the best CD3^+^ T cell binder by flow cytometry. In TCR-based T cell engagers, an aglycan hIgG1-Fc^null^ variant was incorporated to avoid confounding reactivities of residual FcRγIII^+^ PBMC-derived NK cells and monocytes in T cell preparations. Furthermore, adverse effects observed with the trifunctional, Ig-like anti-CD3ε–anti-EpCAM bsAb catumaxomab, incorporating mouse IgG2a-Fc and rat IgG2b-Fc, suggest that T cell retargeting should not be combined with an FcR-binding and ADCC-eliciting Fc part [68]. Upon systemic administration, fatal hepatotoxicity due to cross-linking of T cells with FcR^+^ liver Kupffer cells was observed. We noted major differences in staining efficiencies among the anti-CD3ε scFv tested, suggesting that the tertiary structure of fusion proteins can limit the accessibility of CD3ε binding sites. As the RA14 TCRα-anti-CD3ε(OKT3_HGL_)-Fc^aglyc^ fusion protein showed the strongest binding, it was selected for further experimental evaluation. Similar to the NK cell engaging constructs, T cell responses in co-cultures with peptide-pulsed target cells were dependent on RA14 TCR affinity, fusion protein concentration and pp65 peptide antigen density regarding the functional read-out cytotoxicity/degranulation, T cell activation and proliferation.

Using higher concentrations (>0.6 nM) of RA14 TCRα-anti-CD3ε(OKT3_HGL_)-Fc^null^ fusion proteins, increasing degrees of T cell degranulation, activation and proliferation, and cytotoxicity toward targets preloaded with control peptide were observed. This might be a result of the bivalent CD3 binding, as a few other anti-CD3-bivalent bispecific antibodies were previously reported to induce unspecific T cell activation [69,70], while this was not the case for OKT3 scFv in a bivalent (anti-TAA scFv-Fc^null^-OKT3_HGL_ scFv)_2_ bsAb format [37,38].

Interestingly, the RA14^HAf^ T cell engager induced a slightly greater unspecific T cell response compared to RA14^LAf^, suggesting that affinity maturation of TCR RA14 that only involved TRAV-CDR3 and TRBV-CDR3 residues not directly participating in peptide binding [22] nevertheless may have resulted in reduced peptide specificity, leading to a low degree of peptide-independent T cell activation. This effect was not detectable when analyzing RA14^HAf/LAf^ TCR binding toward survivin peptide-pulsed T2 cells or toward survivin peptide-tethered MHC-Fc fusion proteins by flow cytometry or ELISA that monitor equilibrium binding of sTCR-Fc proteins, suggesting that non-specific activation by survivin/A2 complexes may have been caused by repetitive short-lived immune synapses. Nevertheless, non-specific T cell activation would be manageable using lower concentrations of ≤0.6 nM, which induced an antigen-specific and strong, almost saturated T cell response.

T cell responses in the presence of RA14^HAf^ TCRα-anti-CD3ε(OKT3_HGL_)-Fc^aglyc^ appeared to function superior compared to tetravalent bispecific T cell engagers targeting CD19 (anti-CD19–Fc^aglyc^–OKT3_HGL_) in co-cultures with T2 cells, or targeting EpCAM (anti-EpCAM–Fc^null^–OKT3_HGL_) in co-cultures with MCF-7, respectively, demonstrating the T cell-activating potential of our TCR-Fc T cell engagers. Although the bivalent binding modes to TAA and CD3ε, as well as CD3ε crosslinking as an effector mechanism, are preserved between TCR-based and antibody-based T cell engagers, results have to be interpreted with caution since antigen densities have not been normalized and the OKT3_HGL_ scFv is located in different positions in fusion proteins.

Since costimulation is an important factor in T cell activation, but natural expression of costimulatory ligands is mostly absent from carcinoma cells, we evaluated whether application of a costimulatory bispecific agent could improve T cell activation, as previously demonstrated in different studies for the combination of bispecific antibodies [38,71,72,73]. Indeed, administration of the costimulatory bsAb anti-EpCAM–Fc^null^–anti-CD28 to the co-culture with peptide-pulsed MCF-7 cells starkly improved the performance of the low-affinity RA14 TCR variant, suggesting that a combination therapy might be beneficial depending on available target cell antigens. This costimulatory bsAb was previously shown to be free of agonistic activity and to act only in conjunction with anti-CD3ε-mediated T cell stimulation [38].

The results obtained with NK and T cell-targeting TCR-Fc fusion proteins with suspension cells or adherent target cells were furthermore confirmed in co-cultures with tumor spheroids to analyze more in vivo-like conditions [74,75]. In co-cultures of NK cells or T cells with spheroids of MCF-7 cells transfected with membrane-bound pp65 or survivin peptide-tethered single-chain trimers, different high-affinity NK (TCR-Fc^enh^, TCRα-αNKp46-Fc^enh^, TCRβ-αNKp46-Fc^enh^) and T cell-targeting (TCRα-αCD3ε(OKT3_HGL_)-Fc^null^) fusion proteins induced peptide antigen-specific target cell lysis, NK and T cell degranulation, activation and cytokine release, as well as T cell proliferation. Also in the spheroid tumor model, the NK cell-engaging TCR-Fc performed similarly to or superior to equimolar concentrations of the ADCC-capable mAb trastuzumab, and T cell-engaging TCR-scFv-Fc performed superior to a bispecific anti-EpCAM–Fc^null^–anti-CD3 antibody, previously shown to potently activate cytotoxic T cells in the presence of EpCAM^+^ MCF-7 spheroids [38]. Thus, in the artificial situation of forced overexpression of pMHC-I complexes, TCR-Fc NK and T cell engagers appear to be comparable in efficacy to established ADCC-mediating antibodies or CD3ε-binding bispecific antibodies.

To further validate our findings obtained with sTCR-(scFv)-Fc fusion proteins in the HCMV p65/A2 model systems, we chose two pairs of tumor peptide–reactive A2-restricted TCRs that were previously affinity-matured, the gp100-specific ImmTAC TCR clone IMCgp100 targeting the HLA-A*02:01-bound gp100_280–288_ peptide [18,63] and TCR 1G4 directed against the HLA-A*02:01-bound NY-ESO-1_157–165_ peptide [27], and cloned them into TCR-Fc^enh^ formats. Successful binding to peptide-tethered MHC-Fc fusion proteins and peptide-pulsed T cells demonstrated the functionality of gp100 and NY-ESO-1-specific TCR-Fc and confirmed the expected differences in binding strength between low- and high-affinity TCR variants. As a third example, high- and low-affinity variants of the MART-1 TCR DMF5 recognizing the EAAGIGILTV/A2 complex were engineered as Fc-conjugated NK and T cell engagers. Preliminary experimental results were similar to the NY-ESO-1-specific TCR 1G4.

The IMCgp100^HAf^ TCR performed less efficiently when incorporated in our bivalent Ig-like format, as could be expected from the published difference between affinities of ~30 pM for the gp100 ImmTAC [18] vs. 62 nM for the bivalent Ig-like RA14^HAf^ TCR [22]. We speculate that the different positioning of TRA and TRB chains and of the covalently linked anti-CD3ε scFv in gp100 ImmTAC, as compared to our Ig-like gp100 T cell engager, led to a loss in binding affinity in the latter, but other reasons, such as *N*-glycosylation of protein produced in mammalian cells vs. production in *E. coli* followed by in vitro refolding, may also have contributed to this result, as glycosylation may influence TCR affinity [76]. Even though specific target binding of the gp100 and NY-ESO-1 peptide–reactive TCR constructs was confirmed by ELISA and flow cytometry, in a limited number of experiments, unfortunately none of the TCR-Fc fusion proteins mediated detectable binding to melanoma cell lines previously described as endogenously expressing the target antigens gp100 or NY-ESO-1, respectively [18,48,49,50,51]. TCR-Fc binding to HLA-A2^+^ melanoma cell lines was, however, observed for the gp100- and NY-ESO-specific high-affinity TCR-Fc variants after exogenous loading with peptide.

In experiments with SK-MEL-5 melanoma targets, we had the opportunity to compare the activity of our gp100/A2-reactive T cell engager in the TCRα-OKT3_HGL_-Fc^null^ format with the gp100/A2-specific ImmTAC tebentafusp [18]. Tebentafusp is in clinical use for the treatment of metastatic uveal melanomas and showed modest overall response rates; however, a significant overall survival benefit [77,78,79,80]. Additional ImmTAC molecules reactive with A*02:01-restricted tumor peptides derived from KRAS^G12D^, NY-ESO-1, PRAME, MAGE-A4 and MAGE-8 are in earlier stages of clinical development [16,81]. ImmTac molecules require weekly infusion due to their smaller molecular weight (ca. 75 kDa) compared to IgG antibodies and the absence of an Fc portion enabling recycling via the FcRn IgG receptor. By contrast, our homodimeric sTCR-anti-CD3ε-Fc T cell engager has a molecular weight of ca. 225 kDa, which would provide for an increased in vivo serum half-life [82].

Using freshly isolated, peripheral blood T cells against gp100 peptide-pulsed SK-MEL-5 target cells, gp100^HAf^ TCRα-OKT3_HGL_-Fc^null^ showed comparable or slightly lower levels of cytotoxicity measured by target cell LDH release, CD107a degranulation, TNFα release, and upregulation of early or late activation markers, CD69 and CD25, in comparison to the gp100 ImmTAC molecule, which could not significantly be increased by using a 20-fold increased concentration. When using SK-MEL-5 cells without preloading of HLA-A2 molecules with exogenous cognate peptide ligand, the ImmTAC-gp100 molecule still induced significant levels of cytotoxicity, degranulation, TNFα and IFNγ release as well as activation marker upregulation, whereas our gp100^HAf^ TCRα-OKT3_HGL_-Fc^null^ fusion protein was clearly less sensitive, with only CD170a and CD69 expression being upregulated beyond background levels. Apparently, those T cell responses that required stronger or more sustained activation were below the threshold of detection. Thus, our TCR-scFc-Fc format induced similar responses in the presence of large quantities of cognate pMHC-I complexes, while it was clearly inferior to tebentafusp when targeting naturally processed gp100/A2 complexes, which can be estimated to be in the range of 100 [18].

Since we had, to the best of our knowledge, used the same affinity-matured gp100-reactive TCR sequence present in ImmTAC-gp100, details of the different molecular architectures may have caused the observed differences in functional efficacy. The significant differences in size between the monovalent TRA/UCHT1 scFv-TRB ImmTAC-gp100 and our bivalent (TRB/TRA-OKT3 scFv-Fc)_2_ likely contributed to the lower sensitivity of the latter as larger size and the rigidity of the Fc part of the retargeting molecule may have dampened entry into the immune synapse and T cell activation as suggested by a previous report comparing an ImmTAC-NY-ESO-1 T cell engager with a TCR-Fc-anti-CD3-Fab format [51], and by studies with bispecific antibodies [83,84]. However, as indicated by a study comparing three TCR-monovalent formats with two different TCR specificities produced in CHO cells [85], other parameters such as flexibility and accessibility of fusion protein components rather than the sheer molecular weight seem to matter for T cell activation. Reportedly, an ImmTAC-like TCR-anti-CD3ε(SP34)-scFv format performed worse than a TCR-anti-CD3ε(SP34)-Fab format with two additional Ig-like domains, while a still larger heterodimeric TRB/TRA-Fc//V_L_/V_H_(SP34)-Fc Ig-like format was the least effective [85]. Notably, in that study, cell lines with endogenous MAGE-A3/NY-ESO-1 epitopes could only be killed by T cells that had been strongly preactivated in vitro, while peptide pre-loaded targets were also lysed by non-activated T cells [85]. In addition, our own preliminary results indicate that a 2-fold molar concentration of a monovalent gp100 TCR^HAf^ TRB/TRA-OKT3_HGL_ scFv fusion protein generated by cleavage of the Fc portion showed even diminished levels of T cell cytotoxicity against gp100 peptide-pulsed SK-MEL-5 or MeWo cells. This result would be consistent with the hypothesis that a higher avidity of bivalent TCR-Fc fusion proteins was indeed beneficial for T cell activation. Accordingly, the cleaved monovalent ImmTAC-like fusion protein did not induce cytotoxicity against unpulsed SK-MEL-5 targets, suggesting that the affinity of our gp100 high-affinity TCR-scFv-Fc was not equivalent to ImmTAC-gp100/tebentafusp.

The results shown in this study with three high/low-affinity pairs of TCR-Fc NK and T cell engagers clearly advocate the usage of affinity-matured TCR variants with binding constants being at least in the lower nanomolar range, as variants with wild-type TCR showed very low functional activity in this study. While it seems indispensable for sufficient efficacy of soluble TCR-based NK and T cell engagers, TCR affinity maturation bears, however, the risk of losing selectivity for a particular peptide–MHC-I complex, particularly if mutagenesis involves TCR CDR2 regions that predominantly interact with the MHC molecule, as is given in the affinity-matured gp100/A2-reactive TCR IMC-gp100 (PDB|7PHR). Affinity-matured TCRs in clinical development require derisking by testing against libraries of potentially cross-reactive self peptides [20,21,86]. However, the affinity of the anti-CD3ε scFv also appears to be of major relevance for the functional efficacy of TCR-based T cell engagers, as high anti-CD3 affinities may lead to severe cytokine release and undesired T cell exhaustion, while an intermediate affinity resulted in sustained T cell activation [87,88,89].

We are convinced that TCR-based bispecific agents offer a potential for T and NK cell redirection, as we have provided evidence here that such recombinant adapter molecules can lead to target cell lysis by NK and cytotoxic T cells, promote NK and T cell activation, and stimulate T cell proliferation in an antigen-specific manner. This approach thus enables opening the realm of intracellular peptides presented by MHC-I molecules to NK cells that had so far only been employed to kill tumor cells decorated by ADCC-mediating monoclonal antibodies recognizing cell surface antigens. This study is limited by the present lack of functional validation in vivo and pharmacokinetic data, as well as the lack of in vivo assessment of immunogenicity and safety. Furthermore, the platform of soluble TCR-based NK cell engagers needs to be expanded beyond HLA-A*02:01-restricted TCRs to ensure broad clinical applicability.

Potential safety issues for both TCR-based NK cell and T cell engagers arise from on-target/off-tumor reactivities of the TCR when the same pMHC-I complexes are shared by tumor and healthy tissues [16]. Furthermore, TCRs can also effect off-target reactivities due to cross-reactivities with other pMHC-I complexes and even alloreactivity with non-cognate MHC-I that may result from degenerated pMHC-I recognition [16]. Our TCR-based NK cell engagers lacking anti-CD3 are not expected to significantly activate cytotoxic T cells, and our Fc-silent T cell engagers should not activate NK cells either, thus avoiding redirected lysis of autologous effector cells. Since Fc receptor expression is not limited to NK cells, other innate immune cells such as activated monocytes/macrophages and granulocytes may, however, contribute to cytokine-mediated cytotoxic effects against tumor or healthy cells sharing targeted pMHC-I complexes.

NK cell therapies have the advantage over T cell therapies of being less prone to develop severe toxicities, including cytokine-release syndrome (CRS) and immune effector cell-associated neurotoxicity syndrome (ICANS) due to lower levels of cytokine release by activated NK cells [1,9,10,11]. ADCC-competent Fc parts in antibodies are essential features of NK cell-recruiting antibodies such as rituximab or trastuzumab and are not known to cause overt toxicities [1,9,10,11]. By contrast, for tebentafusp, dose-limiting toxicities have been reported [1,9,10,11], and treatment with tebentafusp is frequently associated with CRS [1,9,10,11], which is caused by a massive and rapid cytokine release by T cells and other cells [1,9,10,11]. Depending on finely balanced antibody affinities, a 2:1 valency of the TAA binder in relation to the CD3 binder has been used to mitigate on-target/off-tumor toxicities in bsAbs [1,9,10,11], and this strategy may as well be applicable to TCR-based T cell engagers.

In this work, the presence of an ADCC-enhanced Fc part was essential for the full functionality of T cell engagers and could not be replaced by an anti-NKp46 scFv or anti-CD16 scFv alone. While the Fc part is dispensable for the function of T cell engagers as demonstrated in ImmTAC molecules, production yields in eukaryotic cells would benefit from its presence. However, as sensitivities were not sufficient to facilitate killing of melanoma targets presenting endogenous epitopes, substantial further efforts are required to improve the molecular architecture of our TCR-Fc-based NK and T cell engagers. The bivalent heterodimeric Fc-containing TCER format [20,21] may represent a good compromise between molecular size reduction and preservation of an IgG1 Fc portion that can be made ADCC-competent.

## 5. Conclusions

In this work, we studied Ig-like TCR-based NK cell engagers containing, as a key functional element, an ADCC-competent Fc part that was complemented by an integrated scFv antibody targeting the activating NK receptor NKp46. T cell engagers were constructed using the same principle but containing an anti-CD3ε scFv and a silenced Fc part. Ig-like TCR-scFv-Fc T cell engagers were less sensitive than a benchmark T cell engager of smaller molecular size; however, they would have the advantage of prolonged serum half-life. Our results emphasize the beneficial role of costimulation provided by bispecific antibodies tethering a tumor antigen to CD28.

## Figures and Tables

**Table 1:** EC_50_ values for cytotoxicity responses of NK cells against MCF-7 target cells with titrated TCR-scFv-Fc proteins or titrated CMV peptide used for MCF-7 preloading shown in Figure 3C.

EC_50_ Values	TCR-(scFv)-Fc Titration [nM]	Peptide Titration [µM]
	Cytotoxicity	
TCR-Fc^enh^	0.119	7.880
TCRβ-anti-CD16-Fc^enh^	0.071	1.953
TCRβ-anti-NKp46-Fc^enh^	0.017	1.699
TCRα-anti-CD16-Fc^enh^	0.064	1.189
TCRα-anti-NKp46-Fc^enh^	0.009	5.879

**Table 2:** EC_50_ values for T cell responses in co-cultures with T2 target cells with titrated TCR-scFv-Fc proteins, as shown in Figure 4C.

EC_50_ Values [nM] (CD3^+^/CD8^+^ T Cells)	TCR-scFv-Fc Titration							
	Cyto-Toxicity	CD25	CD69	CD137	Proliferation	CD107a	IFNγ	TNFα
TCR^Laf^α-anti-CD3-Fc^null^	0.021	0.050	0.010	0.047	0.005	0.020	0.024	0.011
TCR^Haf^α-anti-CD3-Fc^null^	0.006	0.007	0.001	0.006	0.001	0.002	0.005	0.001
anti-CD19-Fc^null^-anti-CD3	0.168	0.036	0.018	0.073	0.051	0.009	0.015	0.014

## Data Availability

The original contributions presented in this study are included in the article/Supplementary Material. Further inquiries can be directed to the corresponding author.

## Supplementary Materials

The following supporting information can be downloaded at: https://www.mdpi.com/article/10.3390/cells15171545/s1, Figure S1: SDS-PAGE analysis of TCR-Fc and TCR-scFv-Fc fusion proteins used in this study; Figure S2: Titration of peptide concentration in co-cultures of peptide-pulsed T2 cells with NK cells in the presence of CMV pp65-specific TCR-Fc fusion proteins; Figure S3: Characterization of tumor cells used as targets for NK cells and T cells with TCR-Fc fusion proteins and bispecifc antibodies; Figure S4: NK cell degranulation and activation by MCF-7 target cells in the presence of TCR-[scFv]-Fc^enh^ fusion proteins; Figure S5: T cell activation by TCR-scFv-Fc fusion protein depends on peptide concentration; Figure S6: T cell activation by TCR-scFv-Fc fusion protein is augmented by high antigen density and costimulation; Figure S7: Co-culture of freshly isolated NK cells with A375 melanoma cells in presence of NY-ESO-1/A2-specific NK cell engagers.

## Abbreviations

The following abbreviations are used in this manuscript:

ADCC	Antibody-dependent cellular cytotoxicity
bsAb	Bispecific antibody
β2m	β2-microglobulin
dtSCT	Disulfide-trapped peptide-tethered MHC-I single-chain trimer
Fc	Fragment crystallizable
MHC-I	Major histocompatibility complex class I antigen
NK	Natural killer cell
PBMC	Peripheral blood mononuclear cell
pMHC-Fc	Peptide MHC class-I complex linked to IgG-Fc
pMHC-mSCT	Plasma membrane-bound peptide-tethered MHC-I single-chain trimer
RM	Repeated-measure
scFv	Single-chain variable fragment antibody
TAA	Tumor-associated antigen
TCR	T cell receptor (antigen-specific)

## References

[1] Klein C., Brinkmann U., Reichert J.M., Kontermann R.E. (2024). The present and future of bispecific antibodies for cancer therapy. Nat. Rev. Drug Discov..

[2] Goebeler M.E., Stuhler G., Bargou R. (2024). Bispecific and multispecific antibodies in oncology: Opportunities and challenges. Nat. Rev. Clin. Oncol..

[3] Jin S., Sun Y., Liang X., Gu X., Ning J., Xu Y., Chen S., Pan L. (2022). Emerging new therapeutic antibody derivatives for cancer treatment. Signal Transduct. Target. Ther..

[4] Shah A., Rauth S., Aithal A., Kaur S., Ganguly K., Orzechowski C., Varshney G.C., Jain M., Batra S.K. (2021). The Current Landscape of Antibody-based Therapies in Solid Malignancies. Theranostics.

[5] Fucà G., Spagnoletti A., Ambrosini M., de Braud F., Di Nicola M. (2021). Immune cell engagers in solid tumors: Promises and challenges of the next generation immunotherapy. ESMO Open.

[6] Middelburg J., Kemper K., Engelberts P., Labrijn A.F., Schuurman J., van Hall T. (2021). Overcoming Challenges for CD3-Bispecific Antibody Therapy in Solid Tumors. Cancers.

[7] Albayrak G., Wan P.K.-T., Fisher K., Seymour L.W. (2025). T cell engagers: Expanding horizons in oncology and beyond. Br. J. Cancer.

[8] Antibody Society. https://www.antibodysociety.org/resources/approved-antibodies/.

[9] Gauthier L., Morel A., Anceriz N., Rossi B., Blanchard-Alvarez A., Grondin G., Trichard S., Cesari C., Sapet M., Bosco F. (2019). Multifunctional Natural Killer Cell Engagers Targeting NKp46 Trigger Protective Tumor Immunity. Cell.

[10] Huan T., Guan B., Li H., Tu X., Zhang C., Tang B. (2023). Principles and current clinical landscape of NK cell engaging bispecific antibody against cancer. Hum. Vaccin. Immunother..

[11] Whalen K.A., Rakhra K., Mehta N.K., Steinle A., Michaelson J.S., Baeuerle P.A. (2023). Engaging natural killer cells for cancer therapy via NKG2D, CD16A and other receptors. mAbs.

[12] Chandran S.S., Klebanoff C.A. (2019). T cell receptor-based cancer immunotherapy: Emerging efficacy and pathways of resistance. Immunol. Rev..

[13] Leko V., Rosenberg S.A. (2020). Identifying and Targeting Human Tumor Antigens for T Cell-Based Immunotherapy of Solid Tumors. Cancer Cell.

[14] Lowe K.L., Cole D., Kenefeck R., OKelly I., Lepore M., Jakobsen B.K. (2019). Novel TCR-based biologics: Mobilising T cells to warm ‘cold’ tumours. Cancer Treat. Rev..

[15] Sun Y., Li F., Sonnemann H., Jackson K.R., Talukder A.H., Katailiha A.S., Lizee G. (2021). Evolution of CD8+ T Cell Receptor (TCR) Engineered Therapies for the Treatment of Cancer. Cells.

[16] Klebanoff C.A., Chandran S.S., Baker B.M., Quezada S.A., Ribas A. (2023). T cell receptor therapeutics: Immunological targeting of the intracellular cancer proteome. Nat. Rev. Drug Discov..

[17] Bossi G., Buisson S., Oates J., Jakobsen B.K., Hassan N.J. (2014). ImmTAC-redirected tumour cell killing induces and potentiates antigen cross-presentation by dendritic cells. Cancer Immunol. Immunother..

[18] Liddy N., Bossi G., Adams K.J., Lissina A., Mahon T.M., Hassan N.J., Gavarret J., Bianchi F.C., Pumphrey N.J., Ladell K. (2012). Monoclonal TCR-redirected tumor cell killing. Nat. Med..

[19] Ribeiro A.R., Britton-Rivet C., Collins L., Carreira R.J., Moureau S., Benlahrech A., Stanhope S., Harper S., Liddy N., Mahon T.M. (2024). High-affinity T cell receptor ImmTAC(R) bispecific efficiently redirects T cells to kill tumor cells expressing the cancer-testis antigen PRAME. Immunother. Adv..

[20] Wermke M., Ochsenreither S., Jaeger D., Becker H., Bleckmann A., Bozorgmehr F., Chatterjee M., Groepper S., Haenel M., Hecker J.S. (2026). MAGE-A4/MAGE-A8-targeted TCR-based bispecific T cell engager in recurrent and/or refractory solid tumors: A phase 1 trial. Nat. Med..

[21] Dilchert J., Hofmann M., Unverdorben F., Kontermann R., Bunk S. (2022). Mammalian Display Platform for the Maturation of Bispecific TCR-Based Molecules. Antibodies.

[22] Wagner E.K., Qerqez A.N., Stevens C.A., Nguyen A.W., Delidakis G., Maynard J.A. (2019). Human cytomegalovirus-specific T-cell receptor engineered for high affinity and soluble expression using mammalian cell display. J. Biol. Chem..

[23] Gras S., Saulquin X., Reiser J.B., Debeaupuis E., Echasserieau K., Kissenpfennig A., Legoux F., Chouquet A., Le Gorrec M., Machillot P. (2009). Structural bases for the affinity-driven selection of a public TCR against a dominant human cytomegalovirus epitope. J. Immunol..

[24] Cox A.L., Skipper J., Chen Y., Henderson R.A., Darrow T.L., Shabanowitz J., Engelhard V.H., Hunt D.F., Slingluff C.L. (1994). Identification of a Peptide Recognized by Five Melanoma-Specific Human Cytotoxic T Cell Lines. Science.

[25] Coughlin C. (2020). Dosing Regimen for GP100-Specific TCR—Anti-CD3 scFv Fusion Protein.

[26] Susac L., Vuong M.T., Thomas C., von Bulow S., O’Brien-Ball C., Santos A.M., Fernandes R.A., Hummer G., Tampe R., Davis S.J. (2022). Structure of a fully assembled tumor-specific T cell receptor ligated by pMHC. Cell.

[27] Robbins P.F., Li Y.F., El-Gamil M., Zhao Y., Wargo J.A., Zheng Z., Xu H., Morgan R.A., Feldman S.A., Johnson L.A. (2008). Single and Dual Amino Acid Substitutions in TCR CDRs Can Enhance Antigen-Specific T Cell Functions. J. Immunol..

[28] Zhao Y., Bennett A.D., Zheng Z., Wang Q.J., Robbins P.F., Yu L.Y.L., Li Y., Molloy P.E., Dunn S.M., Jakobsen B.K. (2007). High-Affinity TCRs Generated by Phage Display Provide CD4+ T Cells with the Ability to Recognize and Kill Tumor Cell Lines1. J. Immunol..

[29] Johnson L.A., Heemskerk B., Powell D.J., Cohen C.J., Morgan R.A., Dudley M.E., Robbins P.F., Rosenberg S.A. (2006). Gene Transfer of Tumor-Reactive TCR Confers Both High Avidity and Tumor Reactivity to Nonreactive Peripheral Blood Mononuclear Cells and Tumor-Infiltrating Lymphocytes. J. Immunol..

[30] Boulter J.M., Glick M., Todorov P.T., Baston E., Sami M., Rizkallah P., Jakobsen B.K. (2003). Stable, soluble T-cell receptor molecules for crystallization and therapeutics. Protein Eng..

[31] Reusch U., Burkhardt C., Fucek I., Le Gall F., Le Gall M., Hoffmann K., Knackmuss S.H., Kiprijanov S., Little M., Zhukovsky E.A. (2014). A novel tetravalent bispecific TandAb (CD30/CD16A) efficiently recruits NK cells for the lysis of CD30+ tumor cells. mAbs.

[32] Arakawa F., Kuroki M., Kuwahara M., Senba T., Ozaki H., Matsuoka Y., Misumi Y., Kanda H., Watanabe T. (1996). Cloning and Sequencing of the VH and YK Genes of an Anti-CD3 Monoclonal Antibody, and Construction of a Mouse/Human Chimeric Antibody. J. Biochem..

[33] Lazar G.A., Dang W., Karki S., Vafa O., Peng J.S., Hyun L., Chan C., Chung H.S., Eivazi A., Yoder S.C. (2006). Engineered antibody Fc variants with enhanced effector function. Proc. Natl. Acad. Sci. USA.

[34] Hale G., De Vos J., Davy A.D., Sandra K., Wilkinson I. (2024). Systematic analysis of Fc mutations designed to reduce binding to Fc-gamma receptors. mAbs.

[35] Tao M.H., Morrison S.L. (1989). Studies of aglycosylated chimeric mouse-human IgG. Role of carbohydrate in the structure and effector functions mediated by the human IgG constant region. J. Immunol..

[36] Lüttgau S., Deppe D., Meyer S., Fertig R., Panjideh H., Lipp M., Schmetzer O., Pezzutto A., Breitling F., Moldenhauer G. (2013). Immunotherapy of B-Cell Lymphoma with an Engineered Bispecific Antibody Targeting CD19 and CD5. Antibodies.

[37] Gonçalves M., Warwas K.M., Meyer M., Schwartz-Albiez R., Bulbuc N., Zörnig I., Jäger D., Momburg F. (2024). Reversal of Endothelial Cell Anergy by T Cell-Engaging Bispecific Antibodies. Cancers.

[38] Warwas K.M., Meyer M., Gonçalves M., Moldenhauer G., Bulbuc N., Knabe S., Luckner-Minden C., Ziegelmeier C., Heussel C.P., Zörnig I. (2021). Co-Stimulatory Bispecific Antibodies Induce Enhanced T Cell Activation and Tumor Cell Killing in Breast Cancer Models. Front. Immunol..

[39] Rölle A., Meyer M., Calderazzo S., Jäger D., Momburg F. (2018). Distinct HLA-E Peptide Complexes Modify Antibody-Driven Effector Functions of Adaptive NK Cells. Cell Rep..

[40] Meyer M., Parpoulas C., Barthelemy T., Becker J.P., Charoentong P., Lyu Y., Börsig S., Bulbuc N., Tessmer C., Weinacht L. (2024). MediMer: A versatile do-it-yourself peptide-receptive MHC class I multimer platform for tumor neoantigen-specific T cell detection. Front. Immunol..

[41] Truscott S.M., Lybarger L., Martinko J.M., Mitaksov V.E., Kranz D.M., Connolly J.M., Fremont D.H., Hansen T.H. (2007). Disulfide Bond Engineering to Trap Peptides in the MHC Class I Binding Groove1. J. Immunol..

[42] Rajendra Y., Kiseljak D., Baldi L., Hacker D.L., Wurm F.M. (2011). A simple high-yielding process for transient gene expression in CHO cells. J. Biotechnol..

[43] Wulhfard S., Baldi L., Hacker D.L., Wurm F. (2010). Valproic acid enhances recombinant mRNA and protein levels in transiently transfected Chinese hamster ovary cells. J. Biotechnol..

[44] Wulhfard S., Tissot S., Bouchet S., Cevey J., De Jesus M., Hacker D.L., Wurm F.M. (2008). Mild hypothermia improves transient gene expression yields several fold in Chinese hamster ovary cells. Biotechnol. Prog..

[45] Liu Z., Chen O., Wall J.B.J., Zheng M., Zhou Y., Wang L., Vaseghi H.R., Qian L., Liu J. (2017). Systematic comparison of 2A peptides for cloning multi-genes in a polycistronic vector. Sci. Rep..

[46] Terrazzano G., Zanzi D., Palomba C., Carbone E., Grimaldi S., Pisanti S., Fontana S., Zappacosta S., Ruggiero G. (2002). Differential involvement of CD40, CD80, and major histocompatibility complex class I molecules in cytotoxicity induction and interferon-γ production by human natural killer effectors. J. Leukoc. Biol..

[47] Boudousquie C., Bossi G., Hurst J.M., Rygiel K.A., Jakobsen B.K., Hassan N.J. (2017). Polyfunctional response by ImmTAC (IMCgp100) redirected CD8(+) and CD4(+) T cells. Immunology.

[48] Chang G.Y., Kohrt H.E., Stuge T.B., Schwartz E.J., Weber J.S., Lee P.P. (2011). Cytotoxic T lymphocyte responses against melanocytes and melanoma. J. Transl. Med..

[49] Ichikawa J., Yoshida T., Isser A., Laino A.S., Vassallo M., Woods D., Kim S., Oelke M., Jones K., Schneck J.P. (2020). Rapid Expansion of Highly Functional Antigen-Specific T Cells from Patients with Melanoma by Nanoscale Artificial Antigen-Presenting Cells. Clin. Cancer Res..

[50] McCormack E., Adams K.J., Hassan N.J., Kotian A., Lissin N.M., Sami M., Mujic M., Osdal T., Gjertsen B.T., Baker D. (2013). Bi-specific TCR-anti CD3 redirected T-cell targeting of NY-ESO-1- and LAGE-1-positive tumors. Cancer Immunol. Immunother..

[51] Zhao W.B., Shen Y., Liu W.H., Li Y.M., Jin S.J., Xu Y.C., Pan L.Q., Zhou Z., Chen S.Q. (2021). Soluble Expression of Fc-Fused T Cell Receptors Allows Yielding Novel Bispecific T Cell Engagers. Biomedicines.

[52] Jones H.F., Molvi Z., Klatt M.G., Dao T., Scheinberg D.A. (2020). Empirical and Rational Design of T Cell Receptor-Based Immunotherapies. Front. Immunol..

[53] Low J.L., Naidoo A., Yeo G., Gehring A.J., Ho Z.Z., Yau Y.H., Shochat S.G., Kranz D.M., Bertoletti A., Grotenbreg G.M. (2012). Binding of TCR multimers and a TCR-like antibody with distinct fine-specificities is dependent on the surface density of HLA complexes. PLoS ONE.

[54] Molldrem J., Zha D. (2024). Unlocking Intracellular Oncology Targets: The Unique Role of Antibody-Based T-Cell Receptor Mimic (TCRm) Therapeutics in T-Cell Engagers (TCEs) and Antibody-Drug Conjugates (ADCs). Cancers.

[55] Haus-Cohen M., Reiter Y. (2024). Harnessing antibody-mediated recognition of the intracellular proteome with T cell receptor-like specificity. Front. Immunol..

[56] Holland C.J., Crean R.M., Pentier J.M., de Wet B., Lloyd A., Srikannathasan V., Lissin N., Lloyd K.A., Blicher T.H., Conroy P.J. (2020). Specificity of bispecific T cell receptors and antibodies targeting peptide-HLA. J. Clin. Investig..

[57] Mosquera L.A., Card K.F., Price-Schiavi S.A., Belmont H.J., Liu B., Builes J., Zhu X., Chavaillaz P.-A., Lee H.-i., Jiao J.-a. (2005). In Vitro and In Vivo Characterization of a Novel Antibody-Like Single-Chain TCR Human IgG1 Fusion Protein. J. Immunol..

[58] Zheng J., Guo Y., Ji X., Cui L., He W. (2013). A novel antibody-like TCRgammadelta-Ig fusion protein exhibits antitumor activity against human ovarian carcinoma. Cancer Lett..

[59] Bethune M.T., Comin-Anduix B., Hwang Fu Y.H., Ribas A., Baltimore D. (2017). Preparation of peptide-MHC and T-cell receptor dextramers by biotinylated dextran doping. Biotechniques.

[60] Farag S.S., Caligiuri M.A. (2006). Human natural killer cell development and biology. Blood Rev..

[61] Bryceson Y.T., March M.E., Ljunggren H.G., Long E.O. (2006). Activation, coactivation, and costimulation of resting human natural killer cells. Immunol. Rev..

[62] Kim H.S., Das A., Gross C.C., Bryceson Y.T., Long E.O. (2010). Synergistic signals for natural cytotoxicity are required to overcome inhibition by c-Cbl ubiquitin ligase. Immunity.

[63] Li Y., Moysey R., Molloy P.E., Vuidepot A.L., Mahon T., Baston E., Dunn S., Liddy N., Jacob J., Jakobsen B.K. (2005). Directed evolution of human T-cell receptors with picomolar affinities by phage display. Nat. Biotechnol..

[64] Croasdale R., Wartha K., Schanzer J.M., Kuenkele K.P., Ries C., Mayer K., Gassner C., Wagner M., Dimoudis N., Herter S. (2012). Development of tetravalent IgG1 dual targeting IGF-1R-EGFR antibodies with potent tumor inhibition. Arch. Biochem. Biophys..

[65] Schanzer J.M., Wartha K., Croasdale R., Moser S., Kunkele K.P., Ries C., Scheuer W., Duerr H., Pompiati S., Pollman J. (2014). A novel glycoengineered bispecific antibody format for targeted inhibition of epidermal growth factor receptor (EGFR) and insulin-like growth factor receptor type I (IGF-1R) demonstrating unique molecular properties. J. Biol. Chem..

[66] Pfeifer Serrahima J., Schoenfeld K., Kühnel I., Harwardt J., Macarrón Palacios A., Prüfer M., Kolaric M., Oberoi P., Kolmar H., Wels W.S. (2024). Bispecific killer cell engagers employing species cross-reactive NKG2D binders redirect human and murine lymphocytes to ErbB2/HER2-positive malignancies. Front. Immunol..

[67] Herault A., Mak J., de la Cruz-Chuh J., Dillon M.A., Ellerman D., Go M., Cosino E., Clark R., Carson E., Yeung S. (2024). NKG2D-bispecific enhances NK and CD8+ T cell antitumor immunity. Cancer Immunol. Immunother..

[68] Borlak J., Länger F., Spanel R., Schöndorfer G., Dittrich C. (2016). Immune-mediated liver injury of the cancer therapeutic antibody catumaxomab targeting EpCAM, CD3 and Fcγ receptors. Oncotarget.

[69] Wu Z., Cheung N.V. (2018). T cell engaging bispecific antibody (T-BsAb): From technology to therapeutics. Pharmacol. Ther..

[70] Bardwell P.D., Staron M.M., Liu J., Tao Q., Scesney S., Bukofzer G., Rodriguez L.E., Choi C.-H., Wang J., Chang Q. (2018). Potent and conditional redirected T cell killing of tumor cells using Half DVD-Ig. Protein Cell.

[71] Hornig N., Reinhardt K., Kermer V., Kontermann R.E., Muller D. (2013). Evaluating combinations of costimulatory antibody-ligand fusion proteins for targeted cancer immunotherapy. Cancer Immunol. Immunother..

[72] Müller D., Frey K., Kontermann R.E. (2008). A Novel Antibody–4-1BBL Fusion Protein for Targeted Costimulation in Cancer Immunotherapy. J. Immunother..

[73] Sapski S., Beha N., Kontermann R., Muller D. (2017). Tumor-targeted costimulation with antibody-fusion proteins improves bispecific antibody-mediated immune response in presence of immunosuppressive factors. Oncoimmunology.

[74] Jeppesen M., Hagel G., Glenthoj A., Vainer B., Ibsen P., Harling H., Thastrup O., Jorgensen L.N., Thastrup J. (2017). Short-term spheroid culture of primary colorectal cancer cells as an in vitro model for personalizing cancer medicine. PLoS ONE.

[75] Weeber F., Ooft S.N., Dijkstra K.K., Voest E.E. (2017). Tumor Organoids as a Pre-clinical Cancer Model for Drug Discovery. Cell Chem. Biol..

[76] Rollins Z., Harris B., George S., Faller R. (2023). A molecular dynamics investigation of *N*-glycosylation effects on T-cell receptor kinetics. J. Biomol. Struct. Dyn..

[77] Middleton M.R., McAlpine C., Woodcock V.K., Corrie P., Infante J.R., Steven N.M., Evans T.R.J., Anthoney A., Shoushtari A.N., Hamid O. (2020). Tebentafusp, A TCR/Anti-CD3 Bispecific Fusion Protein Targeting gp100, Potently Activated Antitumor Immune Responses in Patients with Metastatic Melanoma. Clin. Cancer Res..

[78] Carvajal R.D., Butler M.O., Shoushtari A.N., Hassel J.C., Ikeguchi A., Hernandez-Aya L., Nathan P., Hamid O., Piulats J.M., Rioth M. (2022). Clinical and molecular response to tebentafusp in previously treated patients with metastatic uveal melanoma: A phase 2 trial. Nat. Med..

[79] Hassel J.C., Piperno-Neumann S., Rutkowski P., Baurain J.F., Schlaak M., Butler M.O., Sullivan R.J., Dummer R., Kirkwood J.M., Orloff M. (2023). Three-Year Overall Survival with Tebentafusp in Metastatic Uveal Melanoma. N. Engl. J. Med..

[80] Nathan P., Hassel J.C., Rutkowski P., Baurain J.F., Butler M.O., Schlaak M., Sullivan R.J., Ochsenreither S., Dummer R., Kirkwood J.M. (2021). Overall Survival Benefit with Tebentafusp in Metastatic Uveal Melanoma. N. Engl. J. Med..

[81] Poole A., Karuppiah V., Hartt A., Haidar J.N., Moureau S., Dobrzycki T., Hayes C., Rowley C., Dias J., Harper S. (2022). Therapeutic high affinity T cell receptor targeting a KRASG12D cancer neoantigen. Nat. Commun..

[82] Chiu M.L., Goulet D.R., Teplyakov A., Gilliland G.L. (2019). Antibody Structure and Function: The Basis for Engineering Therapeutics. Antibodies.

[83] Dickopf S., Georges G.J., Brinkmann U. (2020). Format and geometries matter: Structure-based design defines the functionality of bispecific antibodies. Comput. Struct. Biotechnol. J..

[84] Chen W., Yang F., Wang C., Narula J., Pascua E., Ni I., Ding S., Deng X., Chu M.L., Pham A. (2021). One size does not fit all: Navigating the multi-dimensional space to optimize T-cell engaging protein therapeutics. mAbs.

[85] Froning K.J., Sereno A., Huang F., Demarest S.J. (2022). Generalizable design parameters for soluble T cell receptor-based T cell engagers. J. Immunother. Cancer.

[86] Kim G.B., Fritsche J., Bunk S., Mahr A., Unverdorben F., Tosh K., Kong H., Maldini C.R., Lau C., Srivatsa S. (2022). Quantitative immunopeptidomics reveals a tumor stroma–specific target for T cell therapy. Sci. Transl. Med..

[87] Leong S.R., Sukumaran S., Hristopoulos M., Totpal K., Stainton S., Lu E., Wong A., Tam L., Newman R., Vuillemenot B.R. (2017). An anti-CD3/anti-CLL-1 bispecific antibody for the treatment of acute myeloid leukemia. Blood.

[88] Moore G.L., Lee S.-H., Schubbert S., Miranda Y., Rashid R., Pong E., Phung S., Chan E.W., Chen H., Endo N. (2015). Tuning T Cell Affinity Improves Efficacy and Safety of Anti-CD38 × Anti-CD3 Bispecific Antibodies in Monkeys—A Potential Therapy for Multiple Myeloma. Blood.

[89] Robertson I.B., Mulvaney R., Dieckmann N., Vantellini A., Canestraro M., Amicarella F., O’Dwyer R., Cole D.K., Harper S., Dushek O. (2024). Tuning the potency and selectivity of ImmTAC molecules by affinity modulation. Clin. Exp. Immunol..

