# Supplementary material for: T-Cell Receptor Single-Chain Antibody IgG1-Fc Fusion Proteins as Bispecific Engagers for Natural Killer and T Cells
Source: Cells. 2026 Aug 27;15(17):1545. doi: 10.3390/cells15171545 (PMC13564851; doi:10.3390/cells15171545)
Supplement: Supplementary file 1 [file cells-15-01545-s001.zip › cells-4411973-supplementary.pdf]

cells

MDPI

Supplementary Materials

T-cell receptor single-chain antibody IgG1-Fc fusion proteins as bispecific engagers for natural killer and T cells

Annkathrin C. Teschner, Márcia Gonçalves, Marten Meyer, Inka Zörnig, Dirk Jäger and Frank Momburg

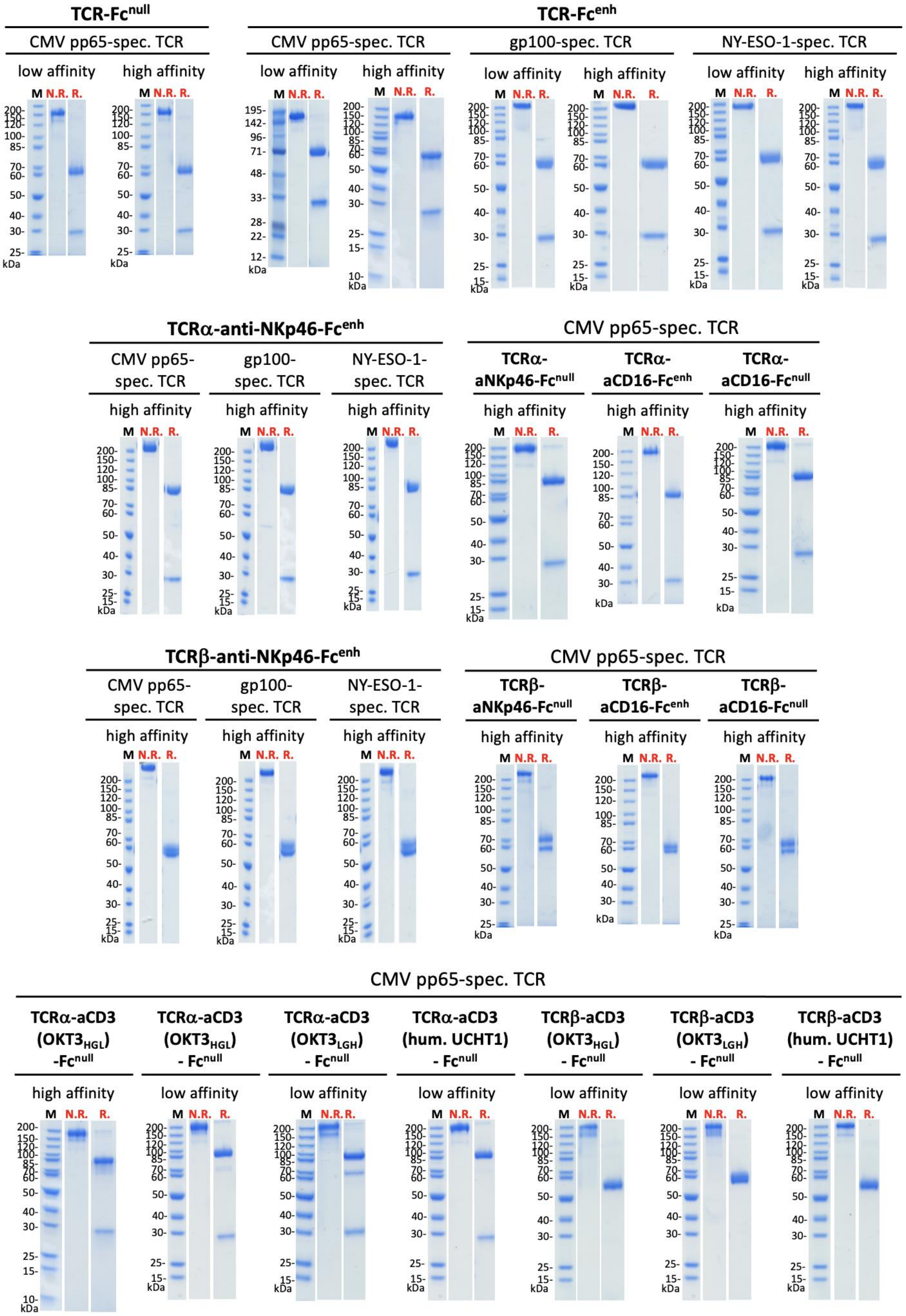

Figure S1. SDS-PAGE analysis of TCR-Fc and TCR-scFv-Fc fusion proteins used in this study. After production in CHO-S and purification by StrepTactin resin, 2.5 μg of dialysed proteins were run in non-reducing or reducing (10 mM DTT) sample buffer on separate gels. Gels were stained with Instant Blue Coomassie protein stain. Lanes from original gel scans were combined according to the identity of the fusion protein. A protein marker is included.

A

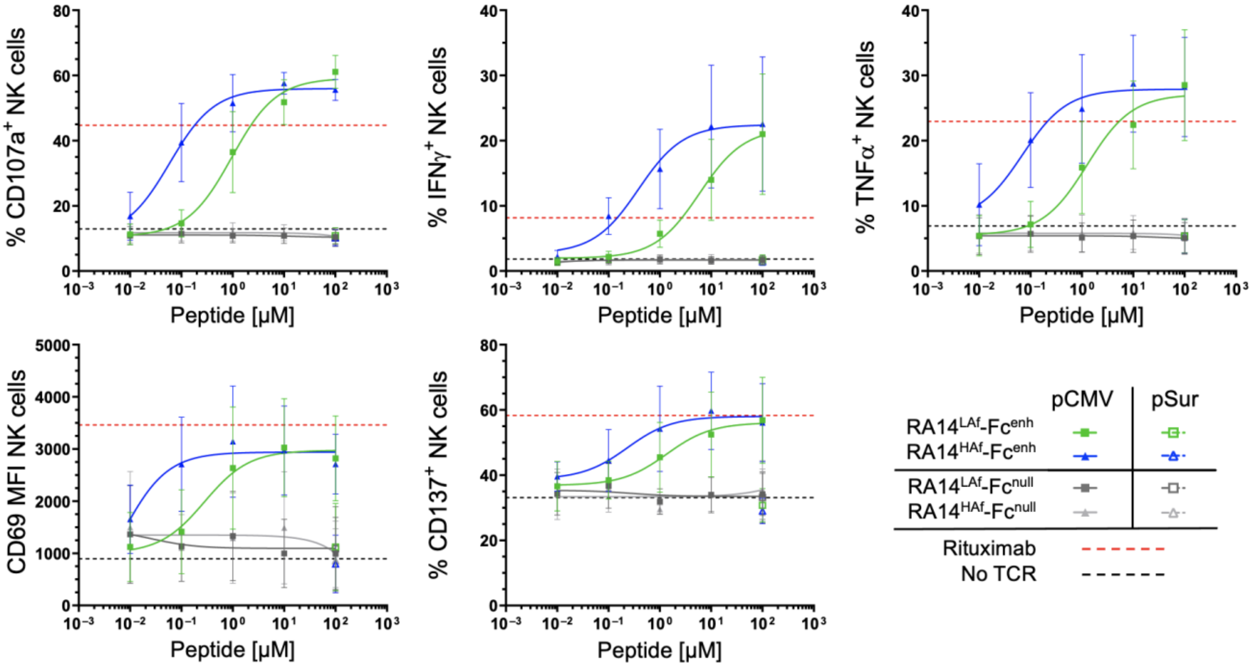

B

T2 target cells

EC50 (µM)	Peptide titration (µM)				
	CD107a	CD137	IFNγ	TNFα	CD69
RA14LAf-Fcenh	0.957	1.362	5.929	1.269	0.280
RA14HAf-Fcenh	0.059	0.232	0.382	0.069	0.007

Figure S2. Titration of peptide concentration in cocultures of peptide-pulsed T2 cells with NK cells in the presence of CMV pp65-specific TCR-Fc fusion proteins. (A) HLA-A2+ T2 cells were preloaded with titrated quantities CMV pp65 peptide NLVPMVATV or survivin peptide LMLGEFLKL for control, and cocultured with freshly isolated NK cells in the presence of 6 nM of TCR RA14$^{LAf/HAf}$-Fc$^{enh/null}$ fusion proteins as indicated. The dotted lines indicate coculture with 6 nM anti-CD20 mAb Rituximab or without TCR-Fc. NK cell degranulation and activation were analyzed using flow cytometry by staining for CD107a, CD69, CD137, and intracellular IFNγ and TNFα. CD107a, IFNγ and TNFα were analyzed after a 4 h coculture in presence of Golgi transport inhibitors. CD69 and CD137 were stained following a 24 h coculture. CD69 having high expression levels on unstimulated NK cells is shown as median fluorescence intensity (MFI). Mean values ± s.e.m. from 3 independent experiments using *n*=3 NK cell donors. (B) EC_{50} values for NK activation with in cocultures with CMV pp65 peptide-pulsed T2 cells from (A).

https://doi.org/10.3390/cells15171545

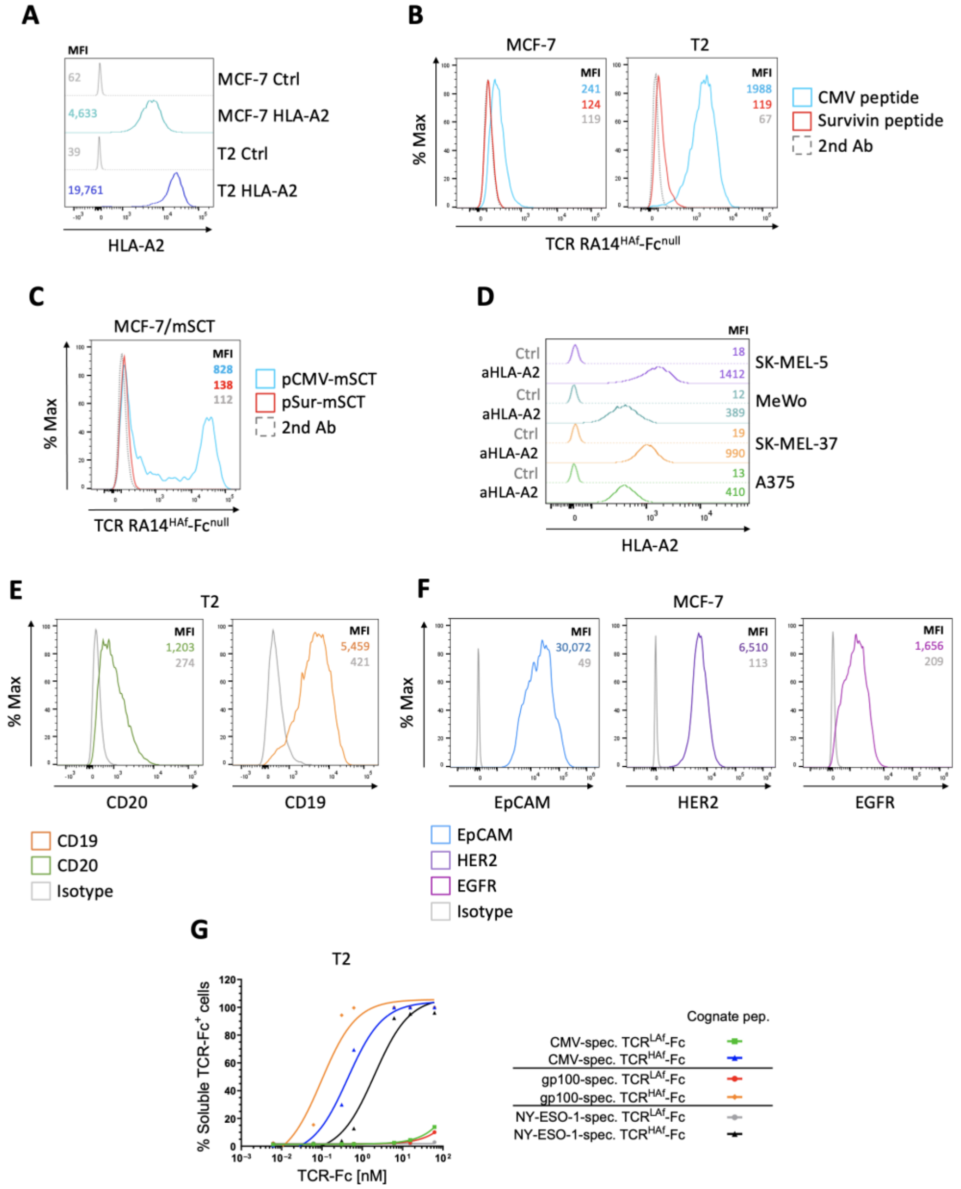

Figure S3. Characterization of tumor cells used as targets for NK cells and T cells with TCR-Fc fusion proteins and bispecific antibodies. (A) Expression levels of HLA-A2 on MCF-7 and T2 cells were analyzed by flow cytometry using PE-conjugated HLA-A2-specific mAb BB7.2 and isotype control. MFI values are indicated. (B) Flow cytometry staining of MCF-7 cells (left) and T2 cells (right) pulsed with 100 μM CMV pp65 peptide or 100 μM survivin peptide by TCR RA14^{HAf}-Fc^{null} fusion protein (6 nM) and PE-conjugated anti-human IgG secondary antibody. (C) Staining of MCF-7 cells transfected with either a membrane-bound HLA-A2 single-chain trimer with tethered CMV pp65 peptide (pCMV-mSCT) or survivin peptide (pSur-mSCT) by TCR RA14^{HAf}-Fc^{null} fusion protein (6 nM) and PE-labeled anti-human IgG secondary antibody. (D) Analysis of HLA-A2 expression levels on melanoma cells by staining flow cytometry using PE-labeled HLA-A2-specific mAb BB7.2 and mouse IgG2b isotype control. MFI values are indicated. (E) Analysis of CD20 (left) and CD19 (right) expression levels on T2 cells by staining flow cytometry using APC-labeled anti-CD20, anti-CD19 and mouse IgG1 isotype control. (F) Analysis of EpCAM (left), HER2 (middle) and EGFR (right) expression levels on MCF-7 cells by staining flow cytometry using APC-labeled anti-EpCAM, PE-labeled anti-HER2, and APC-labeled anti-EGFR antibodies and respective mIgG1 isotype controls (mIgG2b-APC, mIgG1-PE and mIgG1-APC). (G) Binding of titrated amounts of TCR A2/gp100$^{LAf/HAf}$-Fc^{enh} and TCR A2/NY-ESO-1$^{LAf/HAf}$-Fc^{enh} fusion proteins, and TCR RA14$^{LAf/HAf}$-Fc^{enh} for comparison, to T2 cells pulsed with 100 μM of the respective cognate peptides. In the representative experiment, binding was detected by flow cytometry using PE-conjugated anti-human IgG and percentage TCR-Fc^{+} T2 cells above 2nd antibody background is shown.

https://doi.org/10.3390/cells15171545

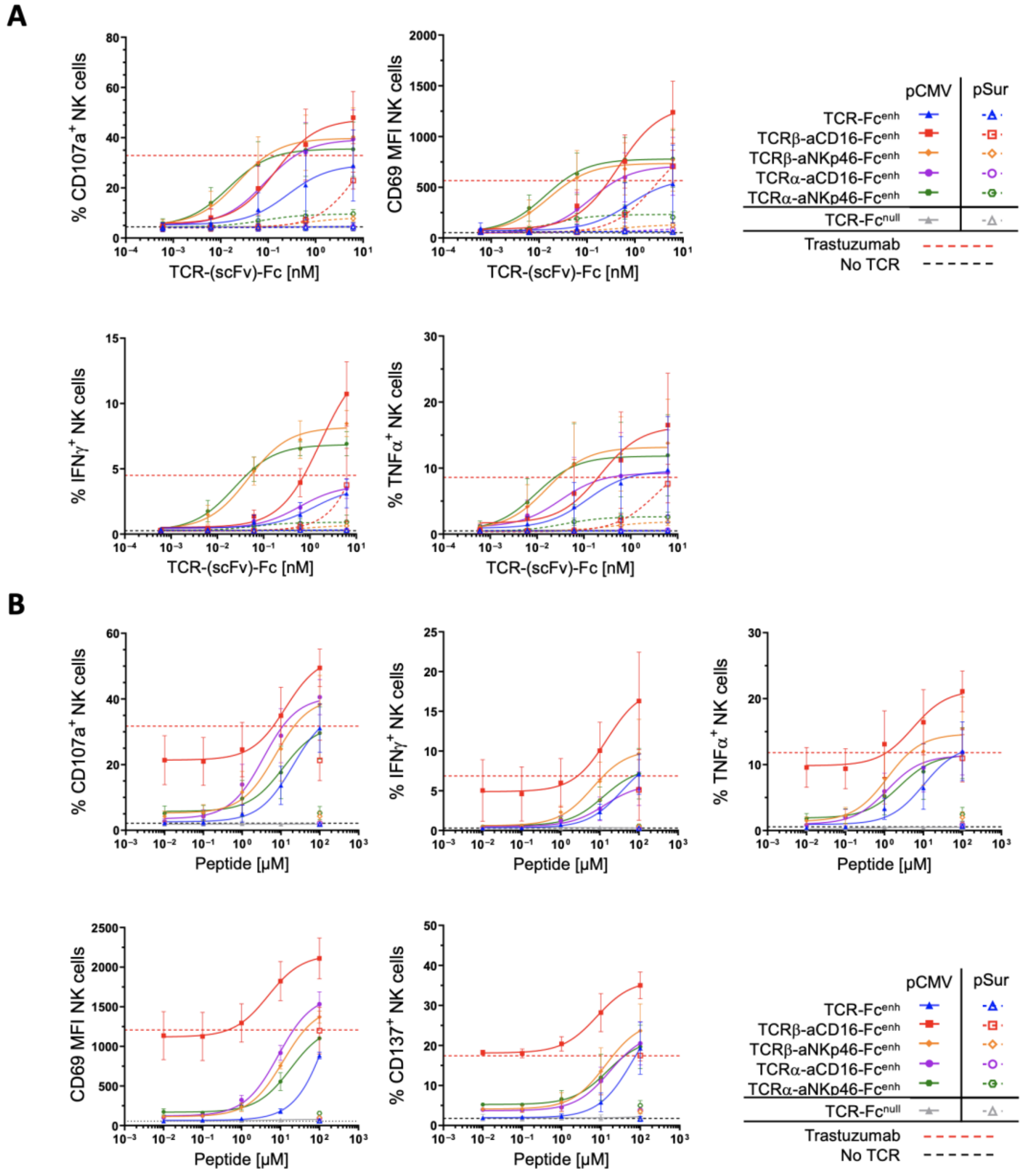

C

EC$_{50}$ (μM) (CD3⁺/CD8⁺ T cells)	TCR titration (nM)				Peptide titration (μM)				
	CD107a	IFNγ	TNFα	CD69	CD107a	IFNγ	TNFα	CD69	CD137
TCR-Fc^{enh}	0.258	1.188	0.119	0.750	20.43	35.50	10.25	199.3	64.40
TCRβ-aCD16-Fc^{enh}	0.143	1.673	0.224	0.452	13.03	15.15	5.379	4.824	7.846
TCRβ-aNKp46-Fc^{enh}	0.023	0.047	0.016	0.016	7.118	6.537	1.194	11.76	13.39
TCRα-aCD16-Fc^{enh}	0.086	0.597	0.032	0.116	3.552	13.19	1.190	8.765	16.74
TCRα–aNKp46-Fc^{enh}	0.015	0.023	0.010	0.013	11.27	12.32	2.437	18.36	14.20

Table title: MCF-7 target cells

Figure S4. NK cell degranulation and activation by MCF-7 target cells in the presence of TCR-[scFv]-Fc^{enh} fusion proteins. (A) Titration of CMV pp65-specific TCR RA14^{HAf}β/α-anti-CD16 scFv-Fc^{enh}, TCR RA14^{HAf}β/α-anti-NKp46 scFv-Fc^{enh} and TCR RA14^{HAf}-Fc^{enh} fusion proteins in cocultures with MCF-7 cells pulsed with 100 μM pCMV (solid lines) or pSur peptides (dashed lines) pulsed and freshly isolated NK cells. TCR RA14^{HAf}-Fc^{null} and no TCR-Fc (straight black line) were included as a negative control. 6 nM ADCC-competent anti-HER2 mAb trastuzumab was included for comparison (straight red line). NK cell degranulation and activation were analyzed using flow cytometry by staining for CD107a, CD69, intracellular IFNγ and TNFα. CD107a, IFNγ and TNFα were evaluated after 4 h co-culture in presence of Golgi transport inhibitors. CD69 was stained following 24 h coculture. Mean values ± s.e.m. from

3 independent experiments with *n*=3 NK cell donors are shown. (B) Titration of pCMV peptide concentration used to preload MCF-7 cells. MCF-7 cells were cocultured with freshly isolated NK cells in the presence of NK cell-engaging CMV pp65-specific TCR $RA14^{HAf}\beta/\alpha$-anti-CD16 scFv-Fc^{enh}, TCR $RA14^{HAf}\beta/\alpha$-anti-NKp46 scFv-Fc^{enh} and TCR $RA14^{HAf}$-Fc^{enh} fusion proteins. Control pSur peptide was only used at the highest concentration (100 μM). TCR-[scFv]-Fc^{enh} fusion proteins were used at 6 nM. TCR $RA14^{HAf}$-Fc^{null} and no TCR-Fc (straight black line) were included as negative controls. 6 nM trastuzumab (straight red line) was applied for comparison. An LDH release assay was performed to evaluate target cell lysis following 4 h co-culture. NK cell degranulation and activation were analyzed using flow cytometry by staining for CD107a, CD69, CD137, intracellular IFNγ and TNFα. CD107a, IFNγ and TNFα were evaluated following 4 h co-culture in presence of Golgi transport inhibitors. CD69 and CD137 were analyzed after 24 h co-culture. Mean values ± s.e.m. from 3 independent experiments with *n*=3 NK cell donors are shown. (C) EC_{50} values obtained from non-linear regression analysis are displayed for the cocultures with NK cells, MCF-7 cells and titrated TCR-[scFv]-Fc^{enh} and titrated peptide corresponding to (A) and (B).

https://doi.org/10.3390/cells15171545

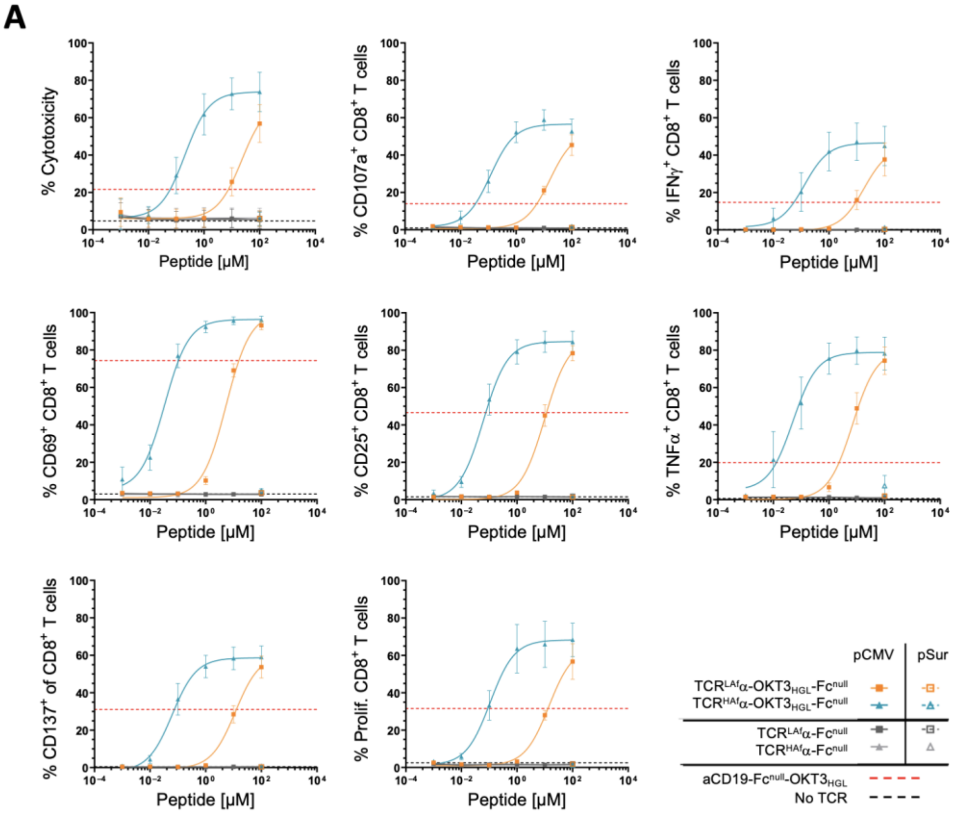

B

T2 target cells

EC$_{50}$ (μM) (CD3$^+$/CD8$^+$ T cells)	Peptide titration							
	Cytotoxicity	CD25	CD69	CD137	Prolife-ration	CD107a	IFNγ	TNFα
TCR^{LAf}α-OKT3$_{HGL}$-Fc^{null}	22.50	9.913	5.421	11.65	14.06	16.27	18.15	7.027
TCR^{HAf}α-OKT3$_{HGL}$-Fc^{null}	0.205	0.061	0.033	0.062	0.108	0.118	0.131	0.05

Figure S5. T cell activation by TCR-scFv-Fc fusion protein depending on peptide concentration. (A) Titration of CMV pp65 peptide (pCMV) concentrations used for preloading T2 cells in coculture of T cells in the presence of 0.06 nM T cell-engaging CMV pp65-specific TCR RA14^{LAf}α-OKT3$_{HGL}$ scFv-Fc^{null} and TCR RA14^{HAf}α-OKT3$_{HGL}$ scFv-Fc^{null} fusion proteins. Control pSur peptide was only used at the highest concentration (100 μM). TCR RA14$^{HAf/LAf}$-Fc^{null} and no TCR-Fc (dashed black line) were included as a negative controls. 0.6 nM anti-CD19-hIgG1-Fc^{null}-anti-CD3ε bsAb (red dashed line) was used for comparison. T cell cytotoxicity was analyzed by LDH release assay after 24 h coculture. Degranulation and activation were analyzed using flow cytometry following 24 h coculture by staining for CD107a, CD25, CD69, CD137, and intracellular IFNγ or TNFα. CD107a, IFNγ and TNFα were evaluated after coculture in presence of Golgi transport inhibitors. T cell proliferation was assessed using CTV-labeled CD3$^+$ T cells as responder cell following 5 days co-culture. Mean values ± s.e.m. from 3 independent experiments using *n*=3 T cell donors are shown. Corresponding EC$_{50}$ values obtained from non-linear regression analysis are listed in (B).

https://doi.org/110.3390/cells15171545

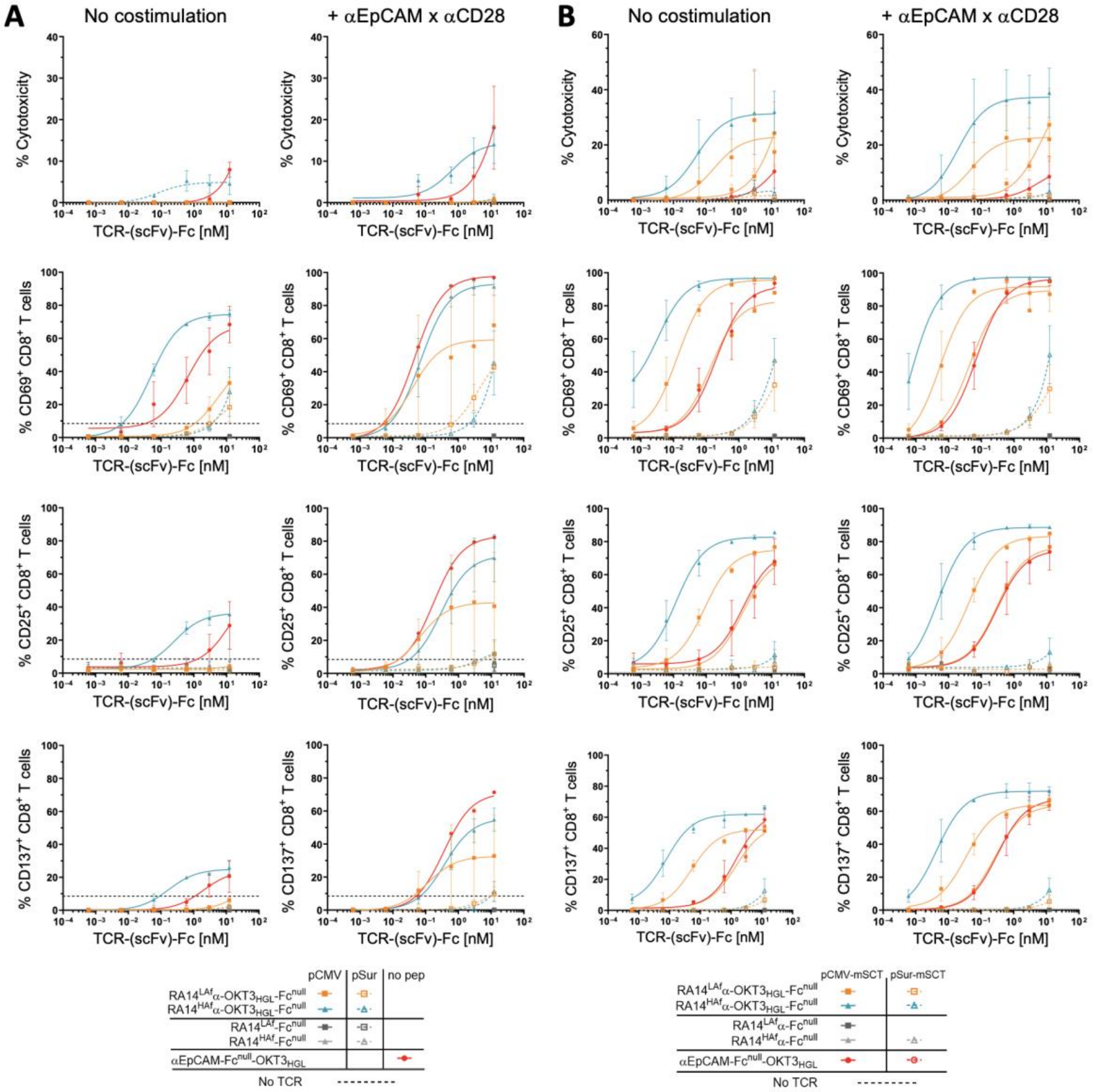

C Peptide-pulsed MCF-7 targets

**EC₅₀ (nM)** (CD3⁺/CD8⁺ T cells)	TCR-scFv-Fc titration (nM) **without** anti-EpCAM-Fc^{null}-anti-CD28				TCR-scFv-Fc titration (nM) **with** 1 nM anti-EpCAM-Fc^{null}-anti-CD28			
	Cyto-toxicity	CD25	CD69	CD137	Cyto-toxicity	CD25	CD69	CD137
TCR^{LAf}α-OKT3$_{HGL}$-Fc^{null}	>100	>100	5.243	47.28	>100	0.065	0.040	0.113
TCR^{HAf}α-OKT3$_{HGL}$-Fc^{null}	0.081	0.256	0.050	0.169	0.642	0.293	0.070	0.381
aEpCAM-Fc^{null}-OKT3$_{HGL}$	>100	12.57	0.663	2.008	25.50	0.172	0.047	0.357

D pCMV-mSCT/CD80-transfected MCF-7 targets

**EC₅₀ (nM)** (CD3⁺/CD8⁺ T cells)	TCR-scFv-Fc titration (nM) **without** anti-EpCAM-Fc^{null}-anti-CD28				TCR-scFv-Fc titration (nM) **with** 1 nM anti-EpCAM-Fc^{null}-anti-CD28			
	Cyto-toxicity	CD25	CD69	CD137	Cyto-toxicity	CD25	CD69	CD137
TCR^{LAf}α-OKT3$_{HGL}$-Fc^{null}	0.170	0.088	0.013	0.052	0.047	0.042	0.005	0.035
TCR^{HAf}α-OKT3$_{HGL}$-Fc^{null}	0.051	0.011	0.003	0.008	0.020	0.005	0.001	0.004
aEpCAM-Fc^{null}-OKT3$_{HGL}$	16.34	1.340	0.200	1.429	6.056	0.301	0.070	0.322

Figure S6. T cell activation by TCR-scFv-Fc fusion protein is augmented by high antigen density and costimulation. (**A**) Titration of TCR RA14^{LAf}α-OKT3$_{HGL}$ scFv-Fc^{null} and TCR RA14^{HAf}α-OKT3$_{HGL}$ scFv-Fc^{null} fusion proteins in cocultures of T cells with MCF-7 cells pulsed with 100 μM pCMV or pSur for antigen-specificity control. TCR RA14$^{HAf/LAf}$-Fc^{null} and no TCR-Fc (dashed black line) were included as negative controls. For comparison, anti-EpCAM-Fc^{null}-anti-CD3ε bsAb was titrated in separate cocultures. Cocultures

were set up with 1 nM of costimulatory anti-EpCAM-Fc^{null}-anti-CD28 bsAb or without. Cytotoxicity was assessed after 24 h coculture by LDH release assay. T cell degranulation and activation were analyzed using flow cytometry following 24 h co-culture by staining for CD107a, CD25, CD69, CD137, and intracellular IFNγ or TNFα. CD107a, IFNγ and TNFα were assessed using cocultures in the presence of Golgi transport inhibitors. T cell proliferation was evaluated by flow cytometry using CTV-labeled T cells following 5 days coculture. Mean values ± s.e.m. from 2 independent experiments are shown. Corresponding EC_{50} values are listed in (**C**). (**B**) Titration of TCR RA14$^{LAf}\alpha$-OKT3$_{HGL}$ scFv-Fc^{null} and TCR RA14$^{HAf}\alpha$-OKT3$_{HGL}$ scFv-Fc^{null} fusion proteins in cocultures of T cells with MCF-7 cells transfected with membrane-bound MHC-I single chain trimers pCMV-mSCT or pCMV-mSur for antigen-specificity control plus CD80. The read-out was conducted as in (**A**). Mean values ± s.e.m. from 2 independent experiments using n=2 T cell donors are shown. Corresponding EC_{50} values are listed in (**D**).

https://doi.org/10.3390/cells15171545

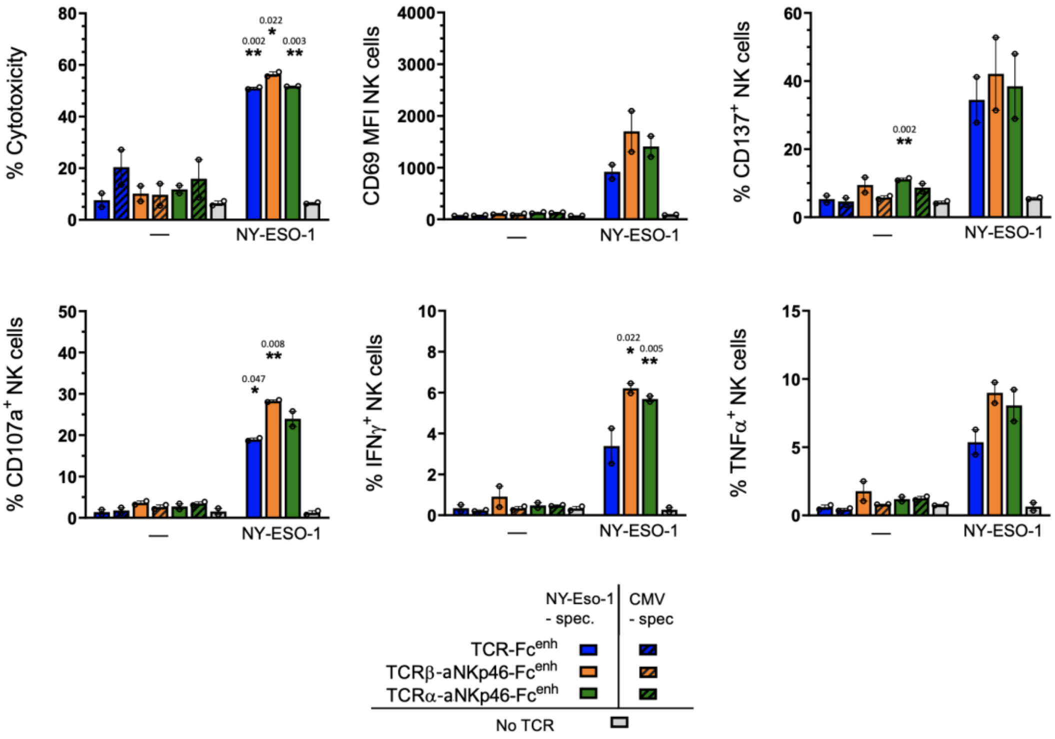

Figure S7. Coculture of freshly isolated NK cells with A375 melanoma cells in presence of NY-ESO-1/A2-specific NK cell engagers. NY-ESO-$1_{157\text{-}165}$/A2-specific TCR-1G4^{HAf}-Fc^{enh} and TCR-1G4^{HAf}β/α-anti-NKp46-Fc^{enh} fusion proteins were used at 6 nM concentration in cocultures with A375 melanoma cells endogenously expressing NY-ESO-1. CMV pp65-specific TCR-RA14^{HAf}-Fc^{enh} and TCR-RA14^{HAf}β/α-anti-NKp46-Fc^{enh} NK cell engagers were included as antigen-specificity controls. A375 cells were pulsed with 100 µM NY-ESO-1 peptide or left untreated. Cytotoxicity was analyzed by LDH release assay following a 4 h coculture. NK cell degranulation and activation were evaluated using flow cytometry by staining for CD107a, CD69, CD137, and intracellular IFNα or TNFα. CD107a, IFNγ and TNFα were analyzed after 4 h coculture in presence of Golgi transport inhibitors. CD69 and CD137 were stained following a 24 h coculture. Significance compared to “No TCR” was analyzed using RM one-way ANOVA test followed by Dunnett’s multiple comparison test. Mean values ± s.e.m. from 2 independent experiments using *n*=2 NK cell donors are shown.

https://doi.org/10.3390/cells15171545
